# Drug cross‐linking electrospun fiber for effective infected wound healing

**DOI:** 10.1002/btm2.10540

**Published:** 2023-05-05

**Authors:** Yuting Luo, Sen Zheng, Kun Wang, Hangqi Luo, Huiling Shi, Yanna Cui, Bingxin Li, Huacheng He, Jiang Wu

**Affiliations:** ^1^ School of Pharmaceutical Sciences, Key Laboratory of Biotechnology and Pharmaceutical Engineering Wenzhou Medical University Wenzhou Zhejiang People's Republic of China; ^2^ College of Chemistry and Materials Engineering Wenzhou University Wenzhou Zhejiang People's Republic of China

**Keywords:** electrospinning, infected wound, molecular simulation, polyvinyl alcohol, tannic acid

## Abstract

The management of infected wounds is still an intractable challenge in clinic. Development of antibacterial wound dressing is of great practical significance for wound management. Herein, a natural‐derived antibacterial drug, tannic acid (TA), was incorporated into the electrospun polyvinyl alcohol (PVA) fiber (TA/PVA fiber, 952 ± 40 nm in diameter). TA worked as a cross‐linker via hydrogen bonding with PVA to improve the physicochemical properties of the fiber and to reach a sustained drug release (88% release of drug at 48 h). Improved mechanical property (0.8–1.2 MPa) and computational simulation validated the formation of the hydrogen bonds between TA and PVA. Moreover, the antibacterial and anti‐inflammatory characteristics of TA laid the foundation for the application of TA/PVA fiber in repairing infected wounds. Meanwhile, in vitro studies proved the high hemocompatibility and cytocompatibility of TA/PVA fiber. Further in vivo animal investigation showed that the TA/PVA fiber promoted the repair of infected wound by inhibiting the bacterial growth, promoting granulation formation, and collagen matrix deposition, accelerating angiogenesis, and inducing M2 macrophage polarization within 14 days. All the data demonstrated that the TA cross‐linked fiber would be a potent dressing for bacteria‐infected wound healing.

## 1 INTRODUCTION

As a global public health problem, skin injury has become one of the leading causes of mortality.^1^, ^2^ Efficient wound repair strategies are urgently needed to accomplish rapid and complete wound healing in clinic. As we know, wound repair is a comprehensive and dynamic course involving four continuous and overlapping phases. Several factors, such as infection and underlying diseases (e.g., diabetes mellitus), have exerted a strong influence on the wound repair, resulting in improper or impaired wound recovery.^3^ Among them, bacterial infection is a typical and serious complication upon wound regeneration.^4^, ^5^, ^6^, ^7^ Once infection occurs, the wound microenvironment becomes complex, resulting in difficulty in healing. For instance, the accumulated reactive oxygen species (ROS) originating from a bacterial infection in the wound bed can elicit significant damage to blood vessels and endothelial cells, inducing strong inflammatory reactions and restraining the functions of macrophages to hinder wound tissue regeneration.^4^, ^8^ Therefore, developing multifunctional antibacterial wound dressing is of great significance to expedite infected wound healing.

In recent years, various novel wound dressings, for example, hydrogels, foams, and films, have been exploited to promote wound repair based on the theory of moist wound healing.^9^, ^10^, ^11^ Among them, the electrospun fibrous dressings have received growing attention with the merits of the porosity, large surface area, flexibility in surface functionalities, and a structure mimicking natural extracellular matrix favoring cell adhesion and proliferation.^12^, ^13^, ^14^, ^15^ Different biocompatible materials have been adopted to fabricate electrospun fibers, among which polyvinyl alcohol (PVA) is an outstanding candidate with good mechanical properties, biodegradability, nontoxicity and excellent hydrophilicity. Previous studies have demonstrated that PVA‐based polymeric nanofibrous scaffold has the capability to accelerate cell proliferation and expedite wound restoration.^16^, ^17^ What is more, PVA is also proved to be a favorable matrix for drug‐loaded filler, which can attain the continuous release of drugs to improve compliance and reduce side effects.^18^, ^19^ Therefore, it can be expected that introducing antibacterial active compounds to electrospun PVA can achieve long‐term antibacterial effects and ultimately promote wound healing.

For wound infection, traditional treatments often involve antibiotics. However, the effectiveness of antibiotics is undermined by drug resistance and the complexity of wound environments.^20^ Here, instead of using antibiotics, we chose the natural broad‐spectrum antimicrobial agents to combat antibacterial infection.^21^ Tannic acid (TA) is a class of natural hydrosoluble polyphenolic compounds widely existing in plants. Recently, it has been proven to be an efficacious antibacterial agent by inhibiting the metabolism of the bacteria.^22^, ^23^, ^24^ Besides, TA also exhibits benign antioxidant, and anti‐inflammatory properties, which makes it quite potent to be employed in wound repair.^25^, ^26^, ^27^ Furthermore, TA with abundant pyrogallol and catechol groups is prone to form hydrogen bonds with other biopolymers (e.g., collagen, PVA, chitosan), laying the foundation for biomedical application.^28^, ^29^, ^30^

Electrospinning is a promising technique that can create functional nanofibers from several directions: large‐scale production^31^; complicated nanostructures, such as core‐shell,^32^ tri‐layer core‐shell,^33^ Janus,^34^ and other multiple‐chamber ones,^35^ and combinations with other chemical and physical methods, such as casting^36^ and cross‐linking.^37^ However, these after‐treatment cross‐linkings are achieved by additional toxic cross‐linkers such as glutaraldehyde.^38^, ^39^ Herein, we carried out a direct cross‐linking of drug‐polymer TA/PVA fibrous membrane (TA/PVA fiber) via hydrogen bonding interaction through electrospinning. First, TA was served as a cross‐linker to stabilize the fiber as well as to obtain a controlled release profile. Meanwhile, TA took the role as both the antibacterial and anti‐inflammatory drug to improve the infected wound repair. The multiple roles of TA were devised and achieved in one simple fibrous membrane, which to our best knowledge had not yet been reported. The obtained electrospun membrane was systematically characterized to investigate the chemical and physical properties. Moreover, in vitro antibacterial property and in vivo infected trauma repair in mice were further explored. The results manifested that TA/PVA fiber could achieve high‐efficient antibacterial effect and promote angiogenesis, granulation formation, and collagen deposition, which eventually hastened infected wound healing.

## 2 MATERIALS AND METHODS

### 2.1 Materials

Polyvinyl alcohol (Mw = ~27,000 Da) and TA (Analytical Reagent) were obtained from Aladdin (Shanghai, China). Hematoxylin and eosin (H&E), anti‐fluorescence quenching agent, dimethylbenzene, 4′,6‐diamidino‐2‐phenylindole (DAPI), ethyl alcohol, carbinol, and resin were purchased from Beyotime Biotech. Inc. (Shanghai, China). DAB (3, 3′‐diaminobenzidine) kit and Masson staining kit were obtained from ZSGB‐BIO (Beijing, China).

### 2.2 Preparation of TA/PVA fiber

Aqueous PVA solution (12%, w/v) was prepared by dispersing 0.6 g PVA in 5 mL ultrapure water and kept continuously stirring at 80°C until complete dissolution. Then, TA was added into the abovementioned PVA solution. After that the mixture was agitated for 2 h. For the electrospinning process, we loaded the homogeneous mixture into a stainless‐steel equipped syringe (10 mL), and then connected the needle to high‐voltage power. Electrospinning was carried out using the following parameters: injection rate = 0.5 mL/h; voltage = 15 kV; the distance from needle tip to collector = 15 cm. The obtained sample was denoted as TA/PVA fiber. Pure PVA fiber was prepared by the same method without the addition of TA.

### 2.3 Characterizations

Crystal structures of the fibers were confirmed using a D8 Advance diffractometer (Bruker, Germany). The morphologies of the samples were visualized under scanning electron microscopy (SEM, Nova 200 NanoSEM, FEI, USA). Infrared spectra were obtained from a Bruker Vector‐22 FTIR spectrophotometer (Germany). Contact angles were measured using a contact angle meter (DSA30, Kruss, Germany). An Instron mechanical tester (3344, Instron, USA) was employed to measure the mechanical strength of the fibers. Thermal analyses were implemented on a DTG‐60H thermogravimetric analyzer (TGA) (Shimadzu, Japan) from 50 to 600°C with the heating speed of 20°C/ min.

### 2.4 Study of TA release profile

The release behavior of TA was studied by detecting its absorbance at λ = 276 nm via the UV–vis spectrophotometer (TU‐1901, China) at various intervals. In a typical procedure, 500 mg of the TA/PVA fiber was submerged in 20 mL of PBS buffer. After given time intervals, 1 mL of the solution was withdrawn and its absorbance was measured. Then 1 mL fresh phosphate‐buffered saline (PBS) buffer was added to the immersion solution, keeping the initial volume unchanged. Every experiment was repeated three times. Meanwhile, to study TA release mechanism from the fiber, the release profile was analyzed based on following mathematical models which include zero‐order model ($\frac{M_{t}}{M_{\infty }}=K_{0}t$), first‐order model ($\log\;M_{t}=\log\;M_{\infty }+\frac{K_{1}t}{2.303}$), Higuchi model ($\frac{M_{t}}{M_{\infty }}=K_{H}t^{0.5}$), and Korsmeyer–Peppas model ($\frac{M_{t}}{M_{\infty }}=K_{p}t^{n}$).^40^, ^41^, ^42^, ^43^ In these models, $M_{t}$ was the cumulative drug amount at time *t*, and $M_{\infty }$ was the drug amount at infinite time, while *K*_0_, *K*_1_, *K*_H_, and *K*_P_ were the respective release constants and *n* denoted diffusion exponent.

### 2.5 Molecular dynamics simulation of PVA‐TA clusters

The system for molecular dynamics (MD) simulation consists of 5 TA molecules and 30 PVA chains with each chain containing 50 repeating units. All the simulations were performed in Gromacs 2021 package,^44^ with the inter‐ and intra‐interactions between TA and PVA molecules represented by the general atom force field (GAFF).^45^ Parameters and topologies for TA and PVA were obtained based on antechamber and ACPYPE tools.^46^ The AM1‐BCC charge model^47^ was used for charging the TA molecule and the MMFF94 charges^48^ were assigned for PVA molecule. An energy minimization was first conducted using the conjugate‐gradient method. Then, the production simulation was completed in the constant‐pressure and constant‐temperature ensemble at fixed pressure (1.01325 bar) and temperature (298.15 K) to obtain the equilibrium structure of PVA‐TA clusters, which ran 10 ns with 2 fs per step. The temperature was controlled by V‐rescale coupling (time constant = 0.1 ps).^49^ The pressure was kept by parrinello‐rahman barostat (coupling constant = 0.5 ps).^50^ PME methods were adopted to determine the electrostatic interactions with the cut‐off distance of 10 Å.^51^ The last 5 ns trajectories were employed for the analysis.

### 2.6 Hemolysis test

To investigate the blood compatibility of the fiber, we conducted a hemolysis assessment. Normal saline (negative) and distilled water (positive) were adopted as the controls. Bloods were firstly cocultured with the saline, deionized water, TA, and TA/PVA fiber. Afterward, the blood was centrifuged, and the supernatants were collected to calculate the hemolysis ratio based on the OD values at 545 nm.

### 2.7 In vitro antibacterial assay

The antimicrobial capability of the fibers was examined through the bacterial colony counting method. In brief, the gram‐positive bacteria, *Staphylococcus aureus (S. aureus)*, were attenuated with saline to achieve a concentration of 10^7^ CFU/mL, and 20 μL bacteria suspension were added into 96‐well plates. Then, 8 mm‐diameter sterilized TA/PVA fibers were added to the 96‐well plate and incubated with the diluted bacteria suspension at 37°C for 2 h. The bacterial suspensions cocultured with 0.9% physiological saline, free TA solutions (low and high) were set as control groups. Subsequently, the bacterial solution was attenuated 1000 times, and then the diluted solution (100 μL) was uniformly plastered onto agar medium. After 24 h incubation at 37°C, the antimicrobial performances were evaluated through counting the bacterial colonies.

### 2.8 In vivo wound healing

The mouse model with an infected wound was applied to assess the antibacterial properties and therapeutic efficacy of TA/PVA fiber. C57BL/6 mice (male, 6–8‐week‐old) were obtained from the Animal Center of Chinese Academy of Sciences (Shanghai, China), and the animal experiments were permitted by the Ethics Committee of Wenzhou Medical University. Animal studies were aligned with the ARRIVE guidelines. Male mice were selected to avoid the hormonal interplay and changes in estrus cycle of females. First, two 8 mm‐diameter wounds on the back of each mouse were created, and 10 μL of *S. aureus* bacterial suspension (10^6^ CFU/mL) was inoculated onto the two wounds. After 48 h, all mice were assigned into four groups (*n* = 8) and received following treatments: saline (control group); free TA solution; PVA fiber (without TA); TA/PVA fiber. The number of mice in each group was determined by G*Power software (version 3.1.9.7, Heinrich Heine Universität Düsseldorf). All treatments were administered for one time. In the end of the experiment (Day 14), PVA fiber and TA/PVA fiber were directly removed from the wounds.

On Days 0, 4, 7, 10, and 14, the appearances of wounds were photographed, and the relative wound sizes (wound healing rate) were analyzed and calculated by ImageJ software. In addition, on Days 2, 4, and 7, bacteria in the wound beds were extracted and incubated on agar plates at 37°C for 24 h to evaluate the in vivo antibacterial activity. On Days 7 and 14, the mice were partially sacrificed to collect the wound tissues for following histological studies.

### 2.9 Histological evaluation

Wound tissues were collected for histological evaluation. Briefly, on the 7th and 14th days, the tissues were carefully collected. After fixation with paraformaldehyde and embedded in paraffin, the slices were stained with H&E and Masson Trichrome. Stained tissue sections were viewed and analyzed using a light microscope (80i, Nikon, Japan). Besides, gram staining was performed on the tissues collected on the 7th day to estimate the in vivo antibacterial effect. On Day 14, the major organs were harvested to access biosafety of the treatments via H&E staining.

### 2.10 Statistical analysis

All results were expressed as mean ± SD from at least triplicated independent experiments. One‐way analysis of variance (ANOVA) combined with Tukey's test were utilized to analyze the statistical significances with GraphPad Prism 8.0 software (GraphPad Software Inc., USA). Statistical significance was set as *p < 0.05 and **p < 0.01, ***p < 0.001.

## 3 RESULTS AND DISCUSSION

### 3.1 Fabrication and characterization of TA/PVA fiber

**FIGURE 1:**
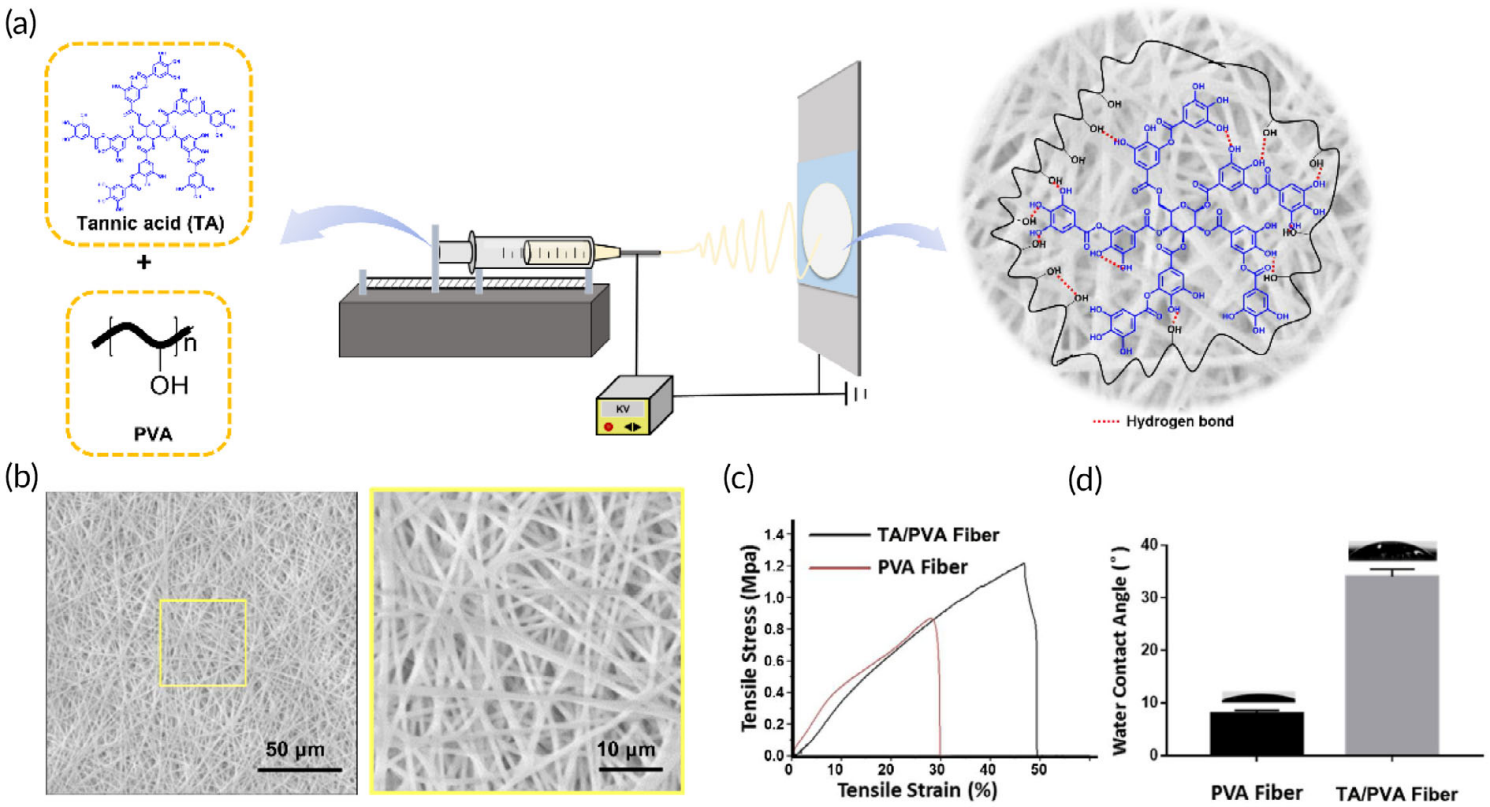
(a) Schematic illustration of the fabrication of tannic acid/polyvinyl alcohol (TA/PVA) fiber by electrospinning. (b) scanning electron microscope (SEM) images of TA/PVA fiber with low and high magnifications. Scale bars: 50 μm (left) and 10 μm (right). (c) Stress–strain curves of PVA fiber and TA/PVA fiber. (d) Water contact angles of PVA fiber and TA/PVA fiber.

Herein, the TA/PVA fiber was prepared via the electrospinning technique. As shown in Figure 1a, both TA and PVA possessed numerous hydroxyl groups, which could interact to form hydrogen bonds to generate a polymer network structure. The morphology of TA/PVA fiber was observed under SEM. The results revealed that the fibers were long and continuous with smooth surfaces and average diameters of 952 ± 40 nm (Figure 1b). Furthermore, we investigated the mechanical property of the fiber. As indicated in Figure 1c, after adding TA, the tensile strength of the fiber increased from 0.8 MPa for PVA fiber to 1.2 MPa for TA/PVA fiber, which was ascribed to the strong hydrogen bonding generated within TA and PVA. In addition, contact angles of the fibers were measured. The contact angle of TA/PVA fiber was 35°, which was higher than the 8° of PVA fiber (Figure 1d). This was quite possible that the generated hydrogen bonding in TA and PVA decreased the amount of free hydrophilic groups (e.g., –OH) in the fibrous structure, which eventually induced the increase of hydrophobicity.^52^ Besides, the swelling ratios of the prepared fibers were also studied. The result found that both PVA fiber and TA/PVA fiber exhibited high swelling abilities (Figure S1), which was beneficial for wound dressing applications to absorb the exudates and create a wet microenvironment to expedite wound recovery. As depicted, the obtained fiber exhibited a downward trend in the swelling ability with the increasing content of TA in the fiber. The main reason presumably was that hydrogen bonding formed between TA and PVA matrix, resulting in the reduction of interactions between O–H groups and water molecules.

**FIGURE 2:**
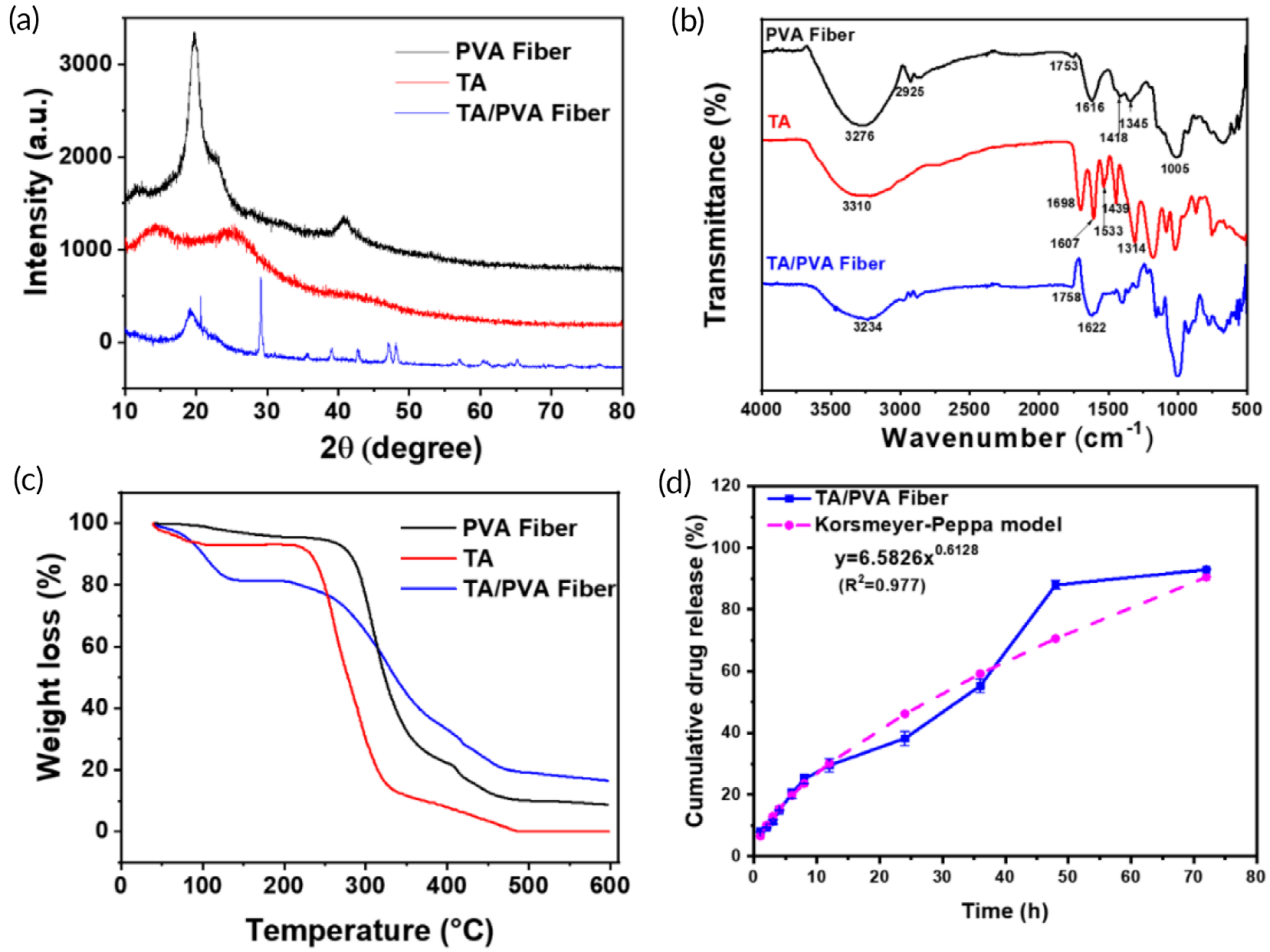
Characterizations of the fibers. (a) X‐ray diffraction (XRD) patterns. (b) Fourier‐transform infrared spectroscopy (FT‐IR) spectra. (c) thermogravimetric analysis (TGA) curves. (d) The cumulative releasing profile and the Korsmetyer–Peppas model fitting curve of tannic acid (TA from TA/polyvinyl alcohol (PVA) fiber.

Figure 2a presented the XRD patterns of free TA, PVA fiber, and TA/PVA fiber. As illustrated, for PVA fiber, typical peaks were found at 2*θ* values of 19.8°, 22.6°, and 40.8°, belonging to the (101), (200), and (102) planes of PVA crystallites.^29^, ^53^ No obvious crystalline peaks were detected for TA, implying the amorphous structure of TA. For TA/PVA fiber, the intensity of typical crystallization peak of PVA was significantly reduced, which was suggested to the formation of hydrogen bonds between TA and PVA.^29^, ^54^, ^55^ Meanwhile, some peaks were found at 2*θ* values of 29°, 47°, and 48.2°, which were originated from the strong interaction in PVA and TA, further confirming the incorporation of TA into the PVA fiber.

Fourier‐transform infrared spectroscopy (FT‐IR) spectra were obtained to confirm chemical bonds in the prepared samples. As illustrated in Figure 2b, the characteristic peak at 3310 cm^−1^ of TA corresponded to the stretching vibration of –OH. Stretching vibration absorption of C=O groups appeared at 1698 cm^−1^. The bands at 1607, 1533, and 1439 cm^−1^ were attributed to the aromatic C—C stretches.^25^, ^26^, ^27^ Besides, the absorption peak at 1314 cm^−1^ is associated with C—O stretching. The peaks ranged from 1000 to 550 cm^−1^ were originated from the bending vibration of the C—H bond on the benzene ring. For PVA fiber, the band at 3276 cm^−1^ was ascribed to ‐OH stretching vibrations and the band at 2925 cm^−1^ was due to stretching vibrations of CH and CH_2_ groups.^56^, ^57^ Moreover, the peaks at 1753, 1413, and 1345 cm^−1^ belonged to C=O, CH_2_, and C—O stretching vibration, respectively, in which the C=O was originated from the residual nonhydrolyzed vinyl acetate groups.^14^, ^58^ In addition, the band at 1616 cm^−1^ was derived from the water absorbed in the fiber. The characteristic peaks of TA/PVA fiber resembled to that of PVA fiber, except that absorption band of hydroxyl stretching vibration exhibited an apparent blue shift to 3234 cm^−1^, implying the formation of the hydrogen bonding in PVA and TA.^56^, ^59^ On account of the generated intramolecular and intermolecular hydrogen bonding, the force constant would decline, consequently resulting in the reduced vibration frequency and blue shift of the –OH stretching vibration.^29^ The apparent blue shift of the FT‐IR results further affirmed the generation of hydrogen bonding in TA and PVA, benefiting for enhanced mechanical strength.

Figure 2c represented the thermal behavior of free TA, PVA fiber, and TA/PVA fiber. For all the samples, the initial weight loss of about 15% occurred below 100°C, which was due to their hydrophilic properties and vaporization that took place in the initial stage. In the second weight loss stage, the temperature ranged from 200 to 500°C. It was worth noting that the initial weight loss temperature of TA/PVA fiber was slightly lower than that of TA and PVA fiber, which might be resulted from the possibility that TA destroyed the crystallization of PVA.^54^, ^60^ All in all, the adequate thermal stability of the TA/PVA film laid the foundation for its application in wound dressings.

Maintaining a steady and continuous release of drug is a vital clinical index for a dressing, as it can reduce the administration frequency and improve patient compliance. Therefore, the release curve of TA was measured. As depicted in Figure 2d, almost 20% of loaded TA was released within the initial 6 h, and then the drug release was continuously proceeded to 55.2% at 24 h and 88% at 48 h. The sustained release of TA was mainly because of the hydrogen bonding interactions. To be noted, the release profile of TA was detected by immersing the TA/PVA nanofibers in PBS, which is different from the situation when used it on the wound bed. As the wound surface is relatively dry, the release time will be longer than the time when soaked in PBS. Furthermore, zero‐order, first‐order, Higuchi and Korsmeyer–Peppas models were employed to assess the TA release kinetics of TA/PVA fiber. For each model, the fitting correlation coefficients of TA release kinetics were calculated and presented in Figure S2. The highest correlation value (*R* ^2^ = 0.9767) suggested that TA release from TA/PVA fiber was well fitted with Korsmeyer–Peppas model. In addition, the *n* value of the TA/PVA fiber in Korsmeyer–Peppas model was >0.5 (*n* = 0.613), which demonstrated the TA release was due to the swelling and relaxation of the fiber.^42^, ^61^

Finally, the degradation ability of the PVA fibers was further studied. The TA/PVA fiber was charged into the top chamber of a transwell, and its bottom surface was in direct contact with the PBS buffer solution in the lower chamber (Figure S3A), which was used to mimic the interaction of the fibrous membrane and the wounded skin. As demonstrated in Figure S3B, even up to 5 days, no obvious degradation of the films was observed as they maintained the same volume. Moreover, the TA/PVA fiber was directly applied to the wound site on the porcine skin to assess the in vivo degradability of the fiber (Figure S3C). Within the 5 days, the fibrous film kept intact in the wound bed without any visual change, suggesting the high stability of the fiber in vivo. Therefore, all these data revealed that TA cross‐linking electrospun PVA fiber was stable.

### 3.2 Hydrogen bonding between PVA and TA

**FIGURE 3:**
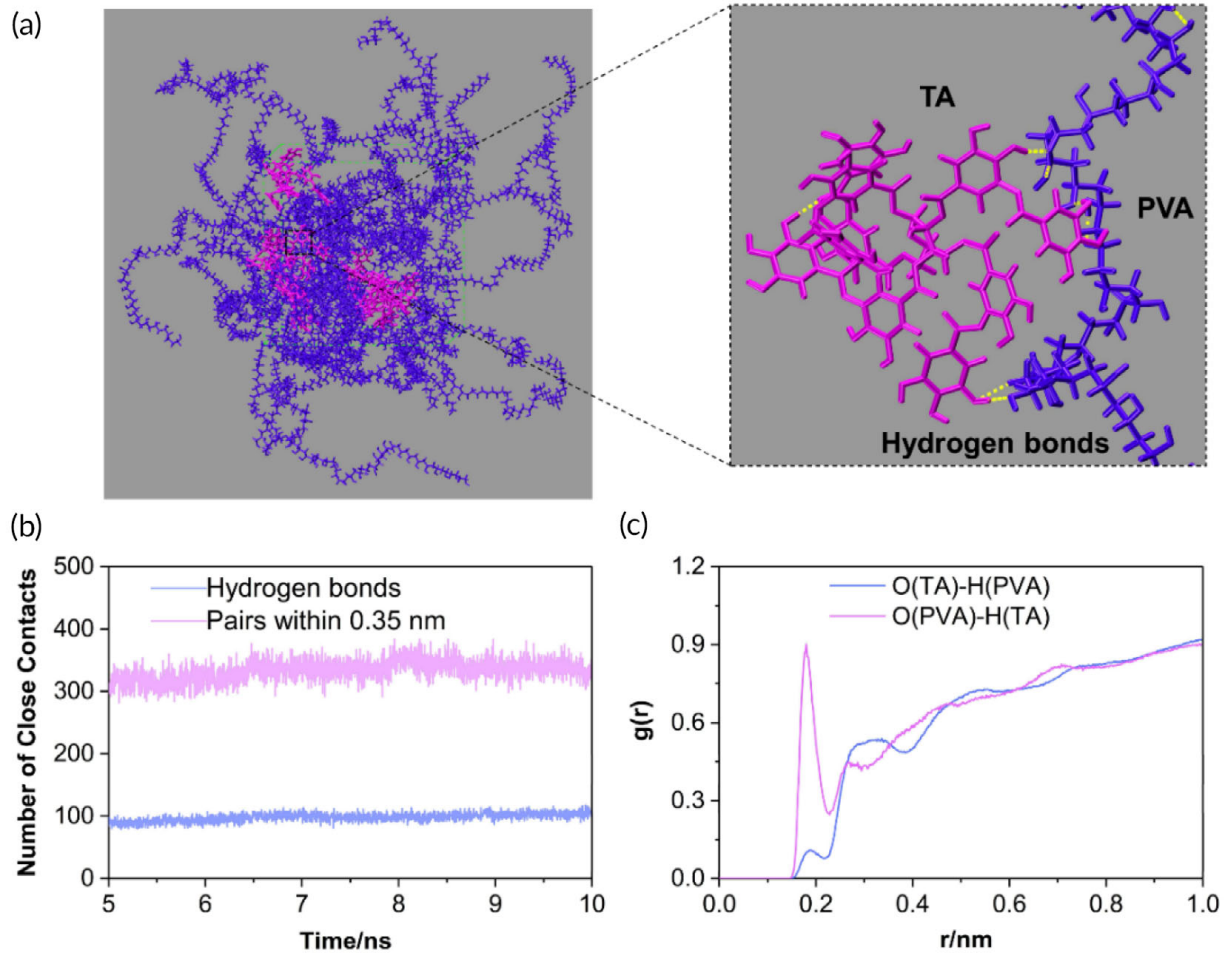
(a) Snapshot of simulated geometrical structure of polyvinyl alcohol/tannic acid (PVA‐TA) and the hydrogen bonds between them. For clarity, the TA molecules were marked magenta and the PVA molecules were marked indigo blue. In the enlarged image, the hydrogen bonds are delineated by the yellow dashed lines. (b) The number of close contacts (pairs within 0.35 nm and hydrogen bonds) between TA and PVA molecules. (c) The radial distribution functions (RDFs) of O—H atomic pairs obtained from the molecular dynamics (MD) trajectory.

Previous studies have manifested that the hydrogen bonds play a significant role in the formation and strengthening of diverse molecular structures.^62^, ^63^ Therefore, the all‐atom MD were performed to verify the presence and dig the details of the hydrogen bonding in TA and PVA. Figure 3a displayed the snapshot of simulated geometrical structure of PVA‐TA with hydrogen bonds formed between them. It could be seen that PVA bore the linear and flexible chain with plenty of hydroxyl groups which could bind with the TA molecules through hydrogen bonds to develop into a three‐dimensional supramolecular cluster. To estimate the interaction power between TA and PVA, the close contacts, including the hydrogen bonds and pairs within 0.35 nm between them, were calculated according to the last 5 ns MD simulations (Figure 3b). There were on average 330 close contacts (defined as the pairs within 0.35 nm) between TA and PVA molecules. Among them, 98 close contacts were recognized as hydrogen bonds, suggesting a high density of hydrogen bonds generated between TA and PVA. In addition, the hydroxyl groups from TA and PVA could act as both hydrogen bond donor and acceptor. Generally, for hydrogen bond X—H⋯A, if the distance of H⋯A was in the range of 1.5–2.2 Å, the interaction was “strong”; and if the distance was in the range of 2.2–3.0 Å, the interaction was “weak.”^64^ With this in mind, the radial distribution function (RDF), which measured the probability density *g*(*r*) of the specified atoms, was employed to further demonstrate the strength of hydrogen bonds. Based on the MD trajectory, the RDF spectra of O (TA)‐H (PVA) and O (PVA)‐H (TA) atomic pairs were calculated and displayed in Figure 3c. The first peak of *g* (*r*) was located at around 1.88 and 1.80 Å for O (TA)‐H (PVA) and O (PVA)‐H (TA) atomic pairs, respectively, suggesting strong hydrogen bonds existing in TA and PVA. In conclusion, these all‐atom MD studies further confirmed the formation of high density of strong hydrogen bonds between TA and PVA molecules.

### 3.3 Biocompatibility and antibacterial ability of TA/PVA fiber

**FIGURE 4:**
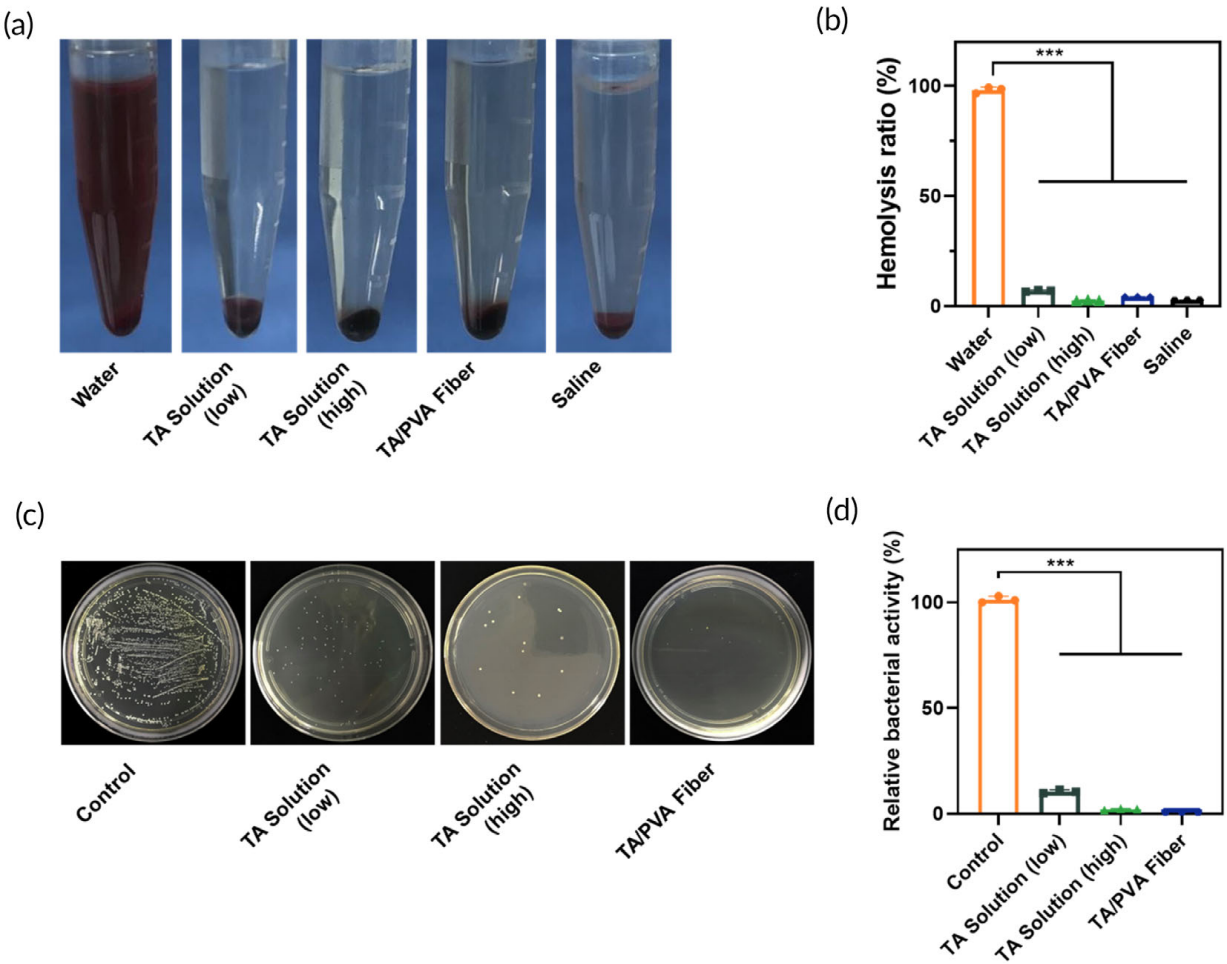
(a) Representative photographs of hemolysis tests of different treatments. The blood was preincubated with water (positive control), tannic acid (TA), TA/polyvinyl alcohol (PVA), or saline (negative control), respectively. (b) Bar diagram of hemolysis ratios after different treatments. (c) Representative images of the colonies formed by *S. aureus* after different treatments. (d) Quantitative results of *S. aureus* colonies after different treatments. Data were stated as mean ± SD, ***p < 0.001, *n* = 3.

Hemocompatibility is one of the key factors of biomaterials' biocompatibility. Quantitative hemolysis ratios of free TA and TA/PVA fiber were determined by hemolysis studies. In Figure 4a,b, the hemolysis ratios of TA/PVA fiber and TA solutions were all less than 5%, which were comparable to that of the saline group. This result indicated that TA/PVA fiber was highly biocompatible and suitable for further in vivo application. What is more, the cytocompatibility of the prepared fiber was studied by MTT assay and live/dead staining using human immortalized epidermal cells (HaCat) and NIH 3T3 (Figure S4). As illustrated in Figure S4A,B, few dead cells (red color) were observed in HaCaT and NIH 3T3 cell lines, suggesting the less cytotoxicity of the treatments. MTT assay further proved that at least 95% cells were alive after 24 and 48 h treatments (Figure S4C–F). Moreover, the host inflammatory response of the PVA fibers was further investigated by subcutaneous implantation experiment. As shown in Figure S5, no detectable host inflammatory response was observed which indicated that the fiber matrix was safe. All the above results indicated that the electrospun fibers systems were highly biocompatible.

For TA is a well‐known broad‐spectrum antibacterial agent, we evaluated the antibacterial efficacy of TA/PVA fiber.^27^, ^65^ The antibacterial properties of PVA fiber, TA solution and TA/PVA fiber against the gram‐positive bacterium *S. aureus* were investigated. As illustrated (Figure 4c,d), TA solution with high TA content and TA/PVA fiber had comparable and considerable antibacterial effect to *S. aureus*. By contrast, TA solution with low concentration displayed reduced antibacterial efficacy. Antibacterial activity testing on TA/PVA fiber was also carried out by disc diffusion method against *S. aureus*, and the inhibition zones were depicted in Figure S6. For TA/PVA fiber, a small circle could be observed surrounding TA/PVA fiber, which further certified the antibacterial property of TA/PVA fiber.

### 3.4 TA/PVA fiber promotes infected wound healing

**FIGURE 5:**
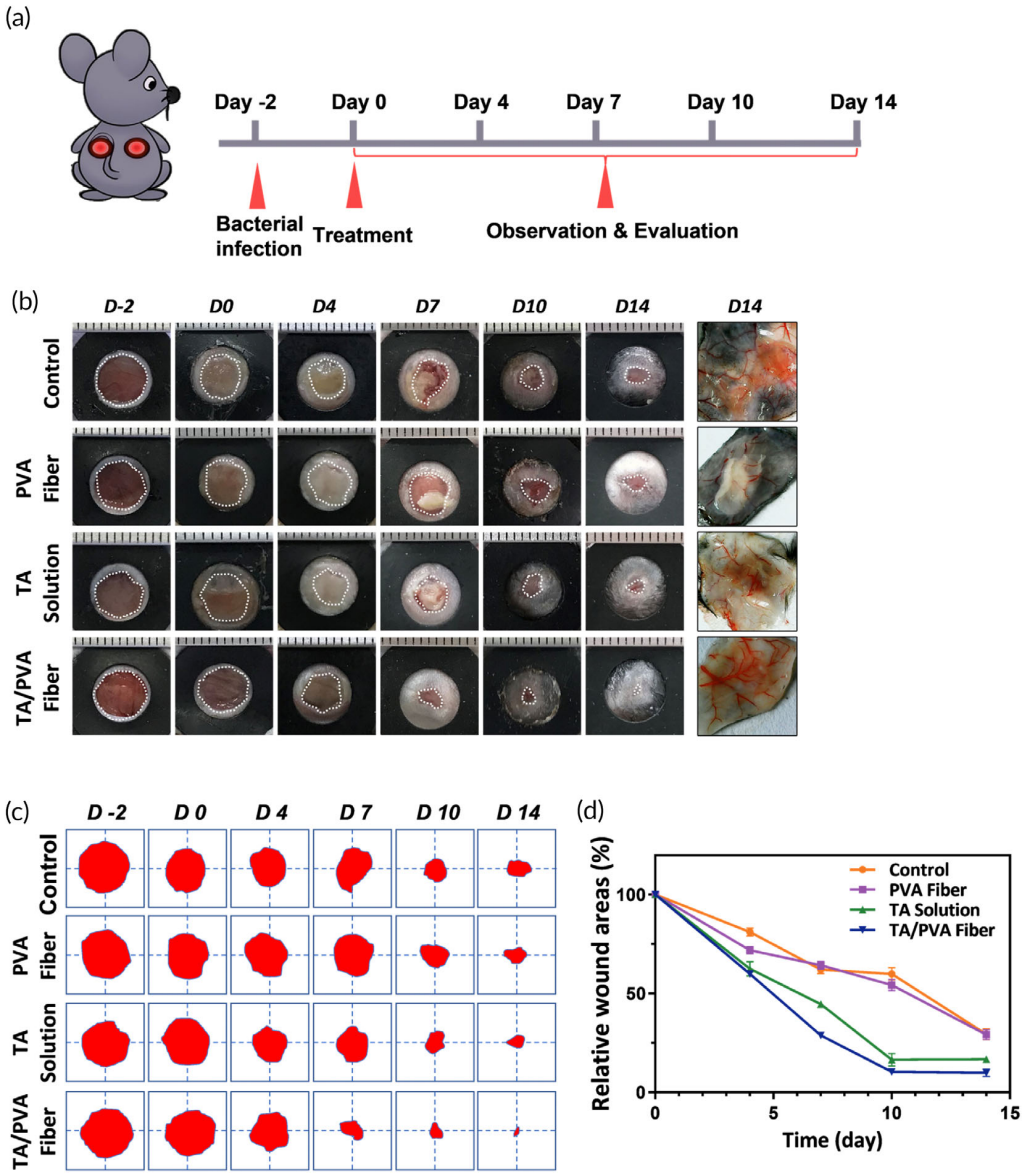
(a) Schematic diagram of the in vivo treatments of phosphate‐buffered saline (PBS (Control)), polyvinyl alcohol (PVA) fiber, tannic acid (TA) solution, and TA/PVA fiber on a mouse model. (b) Representative images of wounds after different treatments. The rightest column: representative dermatoscopic photographs of the skin tissues on Day 14 after different treatments. (c) Wound area traces at different times. The red color area represented the residual wounds. (d) Quantitative analysis of wound areas of different groups.

With the merits of the above physical, chemical, and biocompatible properties, further studies were conducted to assess the in vivo therapeutic efficiency of TA/PVA fiber. As depicted in Figure 5a, the mice with infected wounds were treated with PBS, PVA fiber (without TA encapsulation), TA solution, and TA/PVA fiber, respectively. The images and tracing of the wounds at different times after the treatments were demonstrated in Figure 5b,c, and the statistical wound areas were summarized in Figure 5d. Obviously, the wound treated with TA/PVA fiber exhibited the smallest area on the 7th day and almost complete closure on Day 14, while the remaining wound areas for control, TA solution and PVA fiber were 28%, 16%, and 28%, respectively, on the 14th day. All above data indicated that the healing efficiency of the infected wounds was remarkably accelerated by the TA/PVA fiber. Moreover, on Day 14, the healed skin tissues were carefully harvested and critically examined under a dermatoscope. The result obviously revealed that in comparison with other groups TA/PVA fiber demonstrated enhanced neovascularization with more ordered distribution of capillaries (the rightest column in Figure 5b). This result further validated that TA/PVA had the best wound healing efficacy among all groups.

### 3.5 TA/PVA fiber exhibits improved antibacterial efficacy in vivo

**FIGURE 6:**
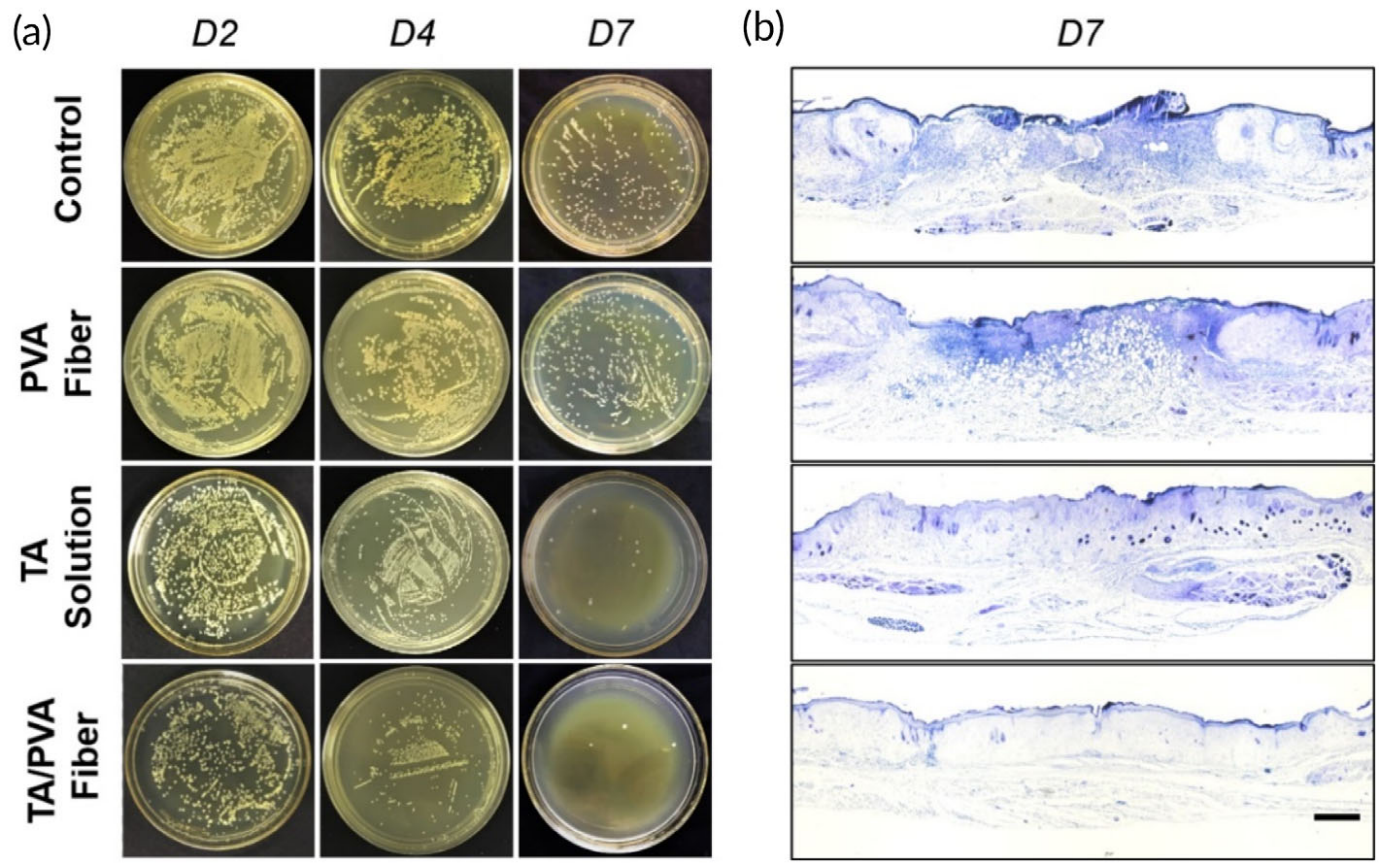
The investigation of the antibacterial effects in vivo. (a) Images of *S. aureus* colonies incubated on agar plates. (b) The gram staining results of wound tissues collected on Day 7. Scale bar: 200 μm.

Moreover, we investigated the in vivo antibacterial property of TA/PVA fiber by monitoring the bacteria amount on the wound beds. As depicted in Figure 6a, PVA fiber group exhibited negligible antibacterial outcome since the number of colonies was comparable to the control group, while TA solution and TA/PVA fiber displayed obvious decrease of colonies from Day 2. Moreover, in comparison with TA solution, TA/PVA fiber showed slightly better elimination effect towards *S. aureus*. This was because of the relatively slow release of TA from the fiber, which helped maintain a higher TA concentration in contrast to its free compartment. Afterwards, gram staining was adopted to observe the distribution of bacteria in the wound sites on Day 7. As shown in Figure 6b, the control and PVA fiber groups exhibited intensive and broad purple distributions, denoting that a great deal of *S. aureus* still resided in the wounds. To the contrary, there were only a few blue distributions existed in the wound sites after treating with TA solution and almost no purple color could be observed in the TA/PVA fiber group. This gram staining result was in consistence with the bacterial colony study, confirming that the TA/PVA fiber could effectively resist bacterial infection in vivo.

### 3.6 TA/PVA fiber facilitates granulation formation, collagen deposition, and maturation

**FIGURE 7:**
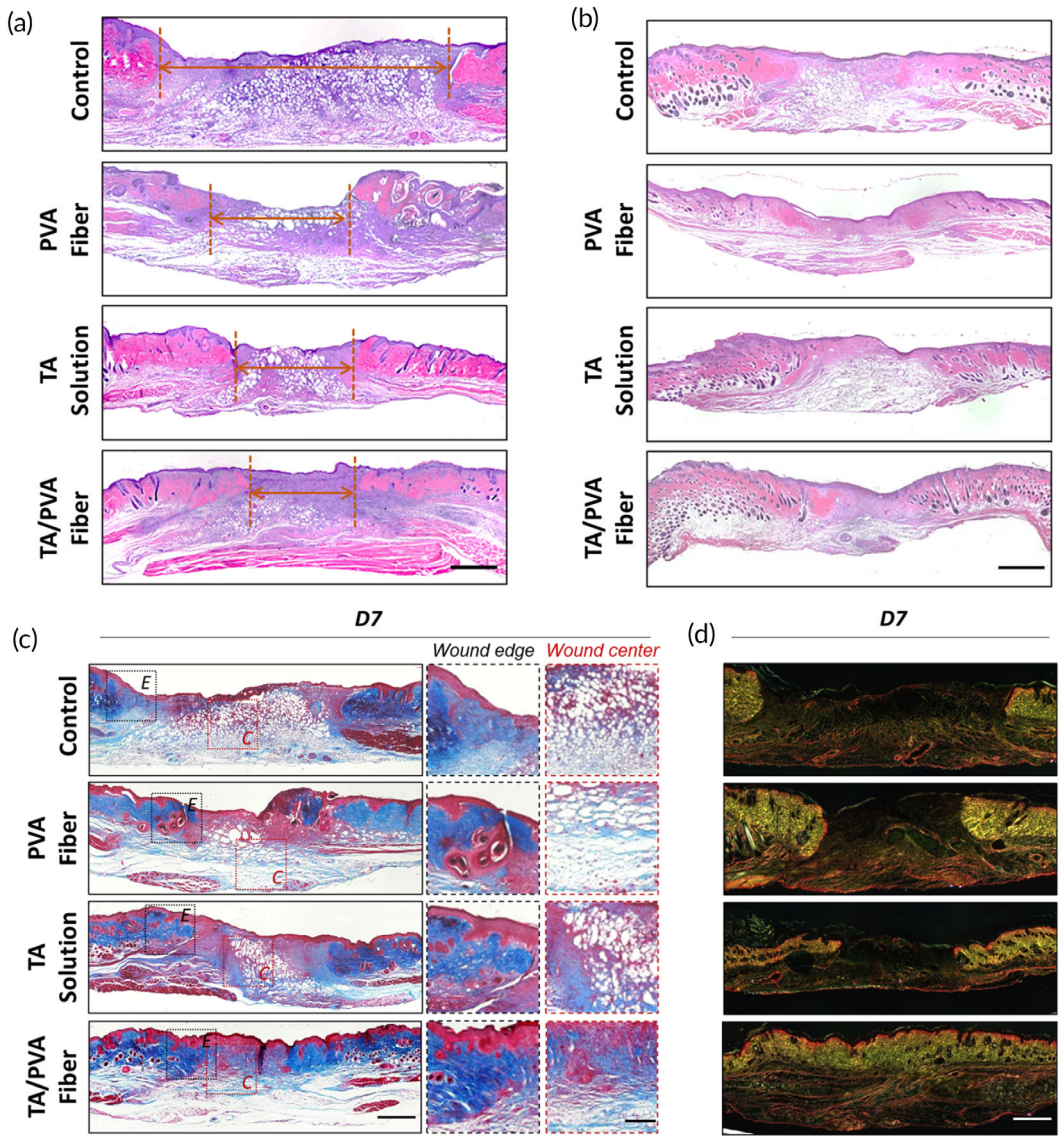
Histological study of wound tissues. Hematoxylin and eosin (H&E) staining results of the wounds on Days 7 (a) and 14 (b). Red arrowheads represented the wound gaps. Scale bar = 1 mm. (c) Masson's trichrome staining result of wounds on Day 7. Both wound edge (E) and wound center (C) were enlarged for better observation. Scale bar for low magnification was 1 mm and 200 μm for high magnification. (d) Picrosirius red (PS) staining result of wounds on Day 7. Scale bar: 1 mm.

To gain the deep inspection of the changes at the wound bed during the wound recovery, the healing performance of TA/PVA fiber was further studied at tissue level via histologic staining. Wound tissues collected on Days 7 and 14 were stained with H&E to observe granulation formation. As displayed in Figure 7a, wounds treated with TA/PVA fiber exhibited the narrowest granulation tissue gap (red arrowhead) on Day 7 compared with other groups, suggesting the faster recovery of wounds after the treatment of TA/PVA fiber. Furthermore, compact granulation tissues as well as newborn epidermis and dermis were visualized within the TA/PVA fiber‐treated group on Day 14, verifying the superior wound repair ability of the TA/PVA fiber (Figure 7b).

Since it was important to determine the tensile strength of the recovered wounds, the collagen deposition in wound beds was further studied by Masson's trichrome staining. In Figure 7c, there were abundant collagen fibers with dark blue color both in the center and edge of the wound sites treated with TA/PVA fiber, indicating that TA/PVA fiber triggered best collagen deposition. What is more, the maturity of the collagen was evaluated by the way of picrosirius red (PS) staining. After PS staining, collagen III corresponding to the initial phase of wound healing was yellow color under polarized light, and collagen I matching the final phase displayed red color. As revealed in Figure 7d, all the groups except TA/PVA fiber demonstrated unhealed wounds with opened epidermis on Day 7, and the collagens in these groups exhibited immatureness with prevalent yellowish color at the wound edges, suggesting the incomplete wound repair. On the contrary, TA/PVA fiber group showed complete epidermis with orange/red color distributed in the wound bed, indicating the fast maturation of the collagen. This result further confirmed that the TA/PVA fiber could restore more mature skin, conforming to H&E and Masson staining results.

### 3.7 TA/PVA fiber enhances angiogenesis

**FIGURE 8:**
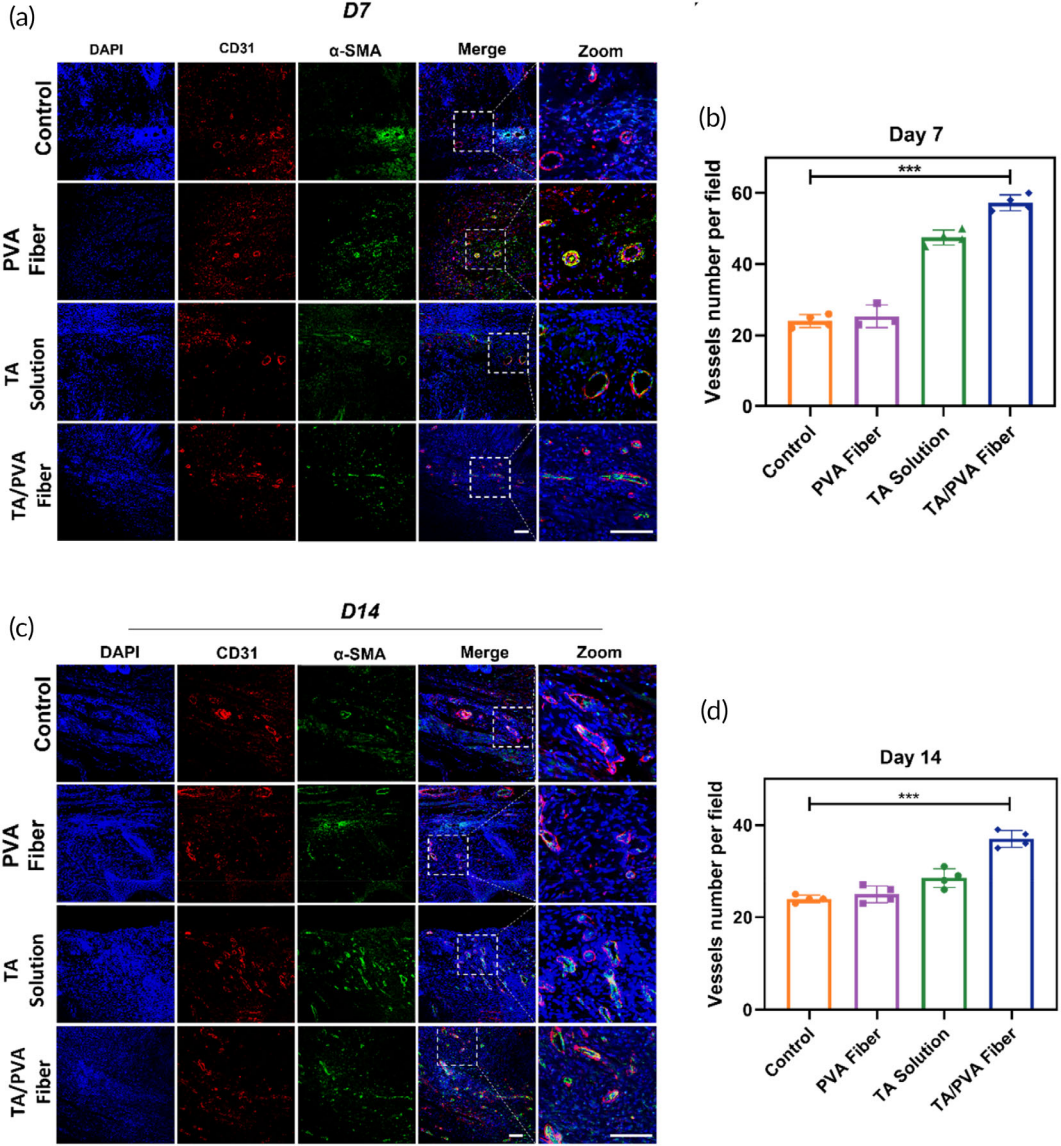
Immunofluorescence staining of CD31 (red) and α‐smooth muscle actin (α‐SMA) (green) of wound tissues treated with PBS (Control), polyvinyl alcohol (PVA) fiber, tannic acid (TA) solution, and TA/PVA fiber on Days 7 (a) and 14 (c). The nuclei were stained as blue by 4′,6‐diamidino‐2‐phenylindole (DAPI). Statistical analysis of blood vessels on Days 7 (b) and 14 (d). Scale bars: 50 μm. Data were given as mean ± SD, ***p < 0.001, *n* = 4.

In the process of wound regeneration, vascular reconstruction is dramatically critical because wound healing activities are extremely relied on the sprouting blood vessels to provide nutrients and oxygen.^66^, ^67^ The immunofluorescent staining of CD31 and α‐SMA, two broadly adopted markers for blood vessels, were adopted to investigate the angiogenesis on Days 7 and 14, respectively. As shown in Figure 8a,c, in comparison with other groups, the TA/PVA fiber group exhibited the most CD31 and α‐smooth muscle actin (α‐SMA) positive spots, suggesting enhanced angiogenesis in the TA/PVA fiber. The statistical result further confirmed that TA/PVA fiber had the best effect to promote the regeneration of blood vessels among all groups (Figure 8b,d). All these demonstrated that TA/PVA fiber could potently enhance vascular endothelial differentiation and angiogenesis, thus, accelerating the wound healing process in the infected wounds.

### 3.8 TA/PVA fiber attenuates inflammation

**FIGURE 9:**
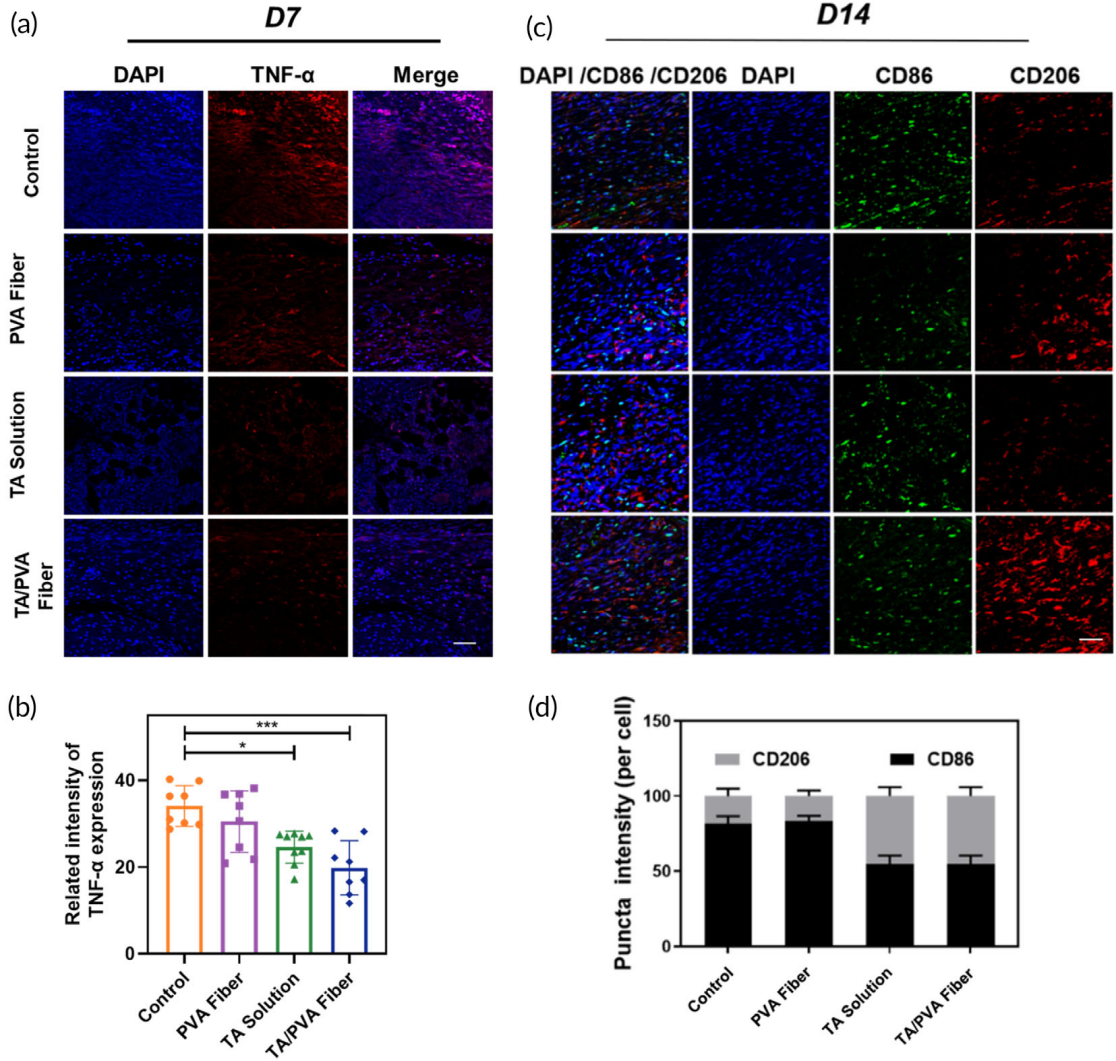
(a) The immunofluorescence staining images of tumor necrosis factor‐α (TNF‐α) (red) of skin wounds on Day 7. The nuclei were stained as blue by 4′,6‐diamidino‐2‐phenylindole (DAPI). Scale bar = 50 μm. (b) Quantitative data of TNF‐α based on the immunofluorescence staining. Data were given as mean ± SD, ***p < 0.001, *n* ≥ 3. (c) Immunofluorescence staining of CD86 (green) and CD206 (red) in wounds with different treatments on Day 14. The nuclei were stained as blue by DAPI. Scale bar = 50 μm. (d) Quantified expressions of CD206 and CD86 in wounds with different treatments on Day 14 (*n* = 3).

Inflammation is a momentous stage in wound healing. However, the prolonged inflammation will delay the wound repair. Thus, the inflammation status of the wounds after TA/PVA fiber treatments was carefully examined to assess the healing efficacy of the fibers. The pro‐inflammatory cytokine, tumor necrosis factor‐α (TNF‐α), was first chosen to explore the anti‐inflammatory activity of the fiber by immunohistochemical analysis. As can be seen from Figure 9a,b, a high secretion of TNF‐α was observed on Day 7 in the wounds treated by PVA fiber, suggesting a severe inflammatory response. On the contrary, much lower expressions of TNF‐α were detected in the wounds treated by TA and TA/PVA fiber, implying the significant attenuation of inflammation in these two groups due to the anti‐inflammatory effect of TA. Furthermore, immunostaining of Ly6G, a marker of neutrophils, was conducted, and the number of neutrophils was quantitatively analyzed. The results shown in Figure S7 demonstrated the less accumulation of neutrophils in the wound bed of TA/PVA treated group, which were in accord with the consequence of TNF‐α staining.

The balance between M1 and M2 macrophage during wound healing was quite important for accelerated wound repair.^68^, ^69^, ^70^ At the beginning of wound healing, M1 macrophage was quickly augmented and dominated in the wound bed, which contributed to the inflammation in the wound bed to remove infections and pathogens. In the repairing stage, M1 macrophage decreased while M2 macrophage significantly increased to accelerate wound healing and remodeling. Therefore, the macrophage polarization and balance were further studied to investigate the healing effect of TA/PVA fiber through the immunofluorescence staining of CD86 and CD206, which were the typical marker of M1 and M2, respectively. As represented in Figure 9c,d, in control and PVA fiber groups, the expression of CD86 was at a relatively high level while CD206 showed low expression, suggesting that the wounds in these groups were still at an early inflammatory stage. In comparison with the control and PVA fiber groups, in both TA solution and TA/PVA fiber groups, the expression of CD86 was reduced while the expression of CD206 was significantly increased. This result indicated that in TA solution and TA/PVA fiber groups M2 macrophage was dominant, implying that the wound healing in these two groups had transited to the repairing stage, which was remarkably improved in comparison with the control and PVA fiber groups. These data collectively proved that the incorporation of TA in TA/PVA fiber endowed the fiber with anti‐inflammatory ability, which eventually accelerated wound repair through regulating M1/M2 macrophage balance.

### 3.9 In vivo biosafety

**FIGURE 10:**
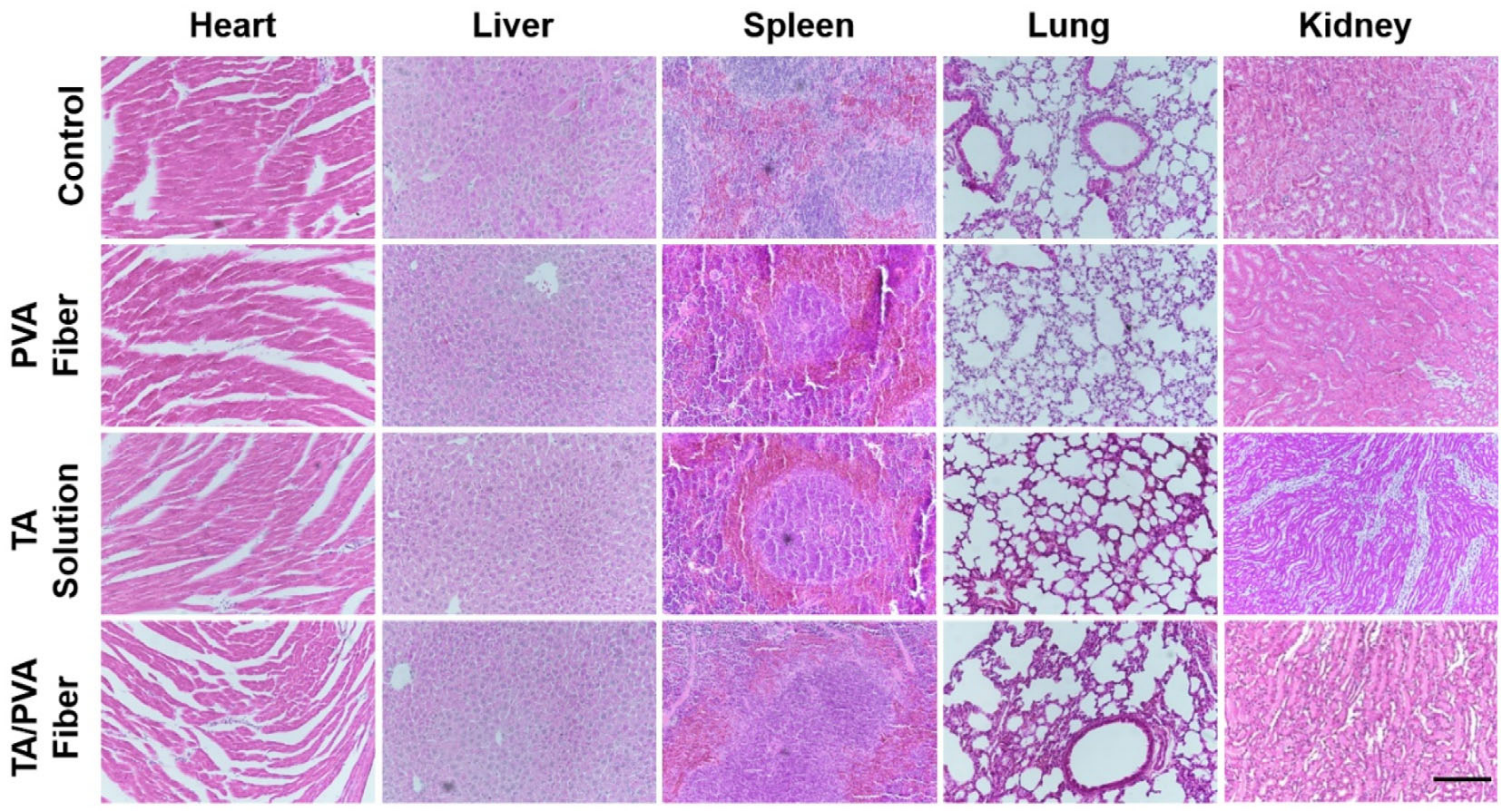
Hematoxylin and eosin (H&E) staining images for major organ tissues on Day 14 after various treatments. Scale bar = 200 μm.

Finally, the biosafety of the TA/PVA fiber in vivo was examined. The mice were sacrificed on Day 14 and the major organs were collected for H&E staining. The result depicted in Figure 10 suggested that there was no abnormal effects or damage with these treatments, certifying that the TA/PVA fiber was a safe therapeutic approach for wound repair.

## 4 CONCLUSIONS

In this work, a TA cross‐linked PVA fiber (TA/PVA fiber) was prepared via the electrospinning method and adopted as a wound healing dressing. Both characterizations and computational simulation demonstrated that the cross‐linking was acquired through the hydrogen bonds between TA and PVA, which improved the physiochemical properties of the fiber and endowing the fiber a sustained TA‐releasing merit. In vitro experiments discovered that the TA/PVA fiber possessed benign hemocompatibility and antibacterial activity. In vivo results manifested that TA/PVA fiber exerted effective prevention of bacterial growth and facilitated wound repair through enhanced granulation formation, collagen deposition, angiogenesis, and M2 macrophage polarization. All these results proved that TA/PVA fiber was a promising alternative as the antiinfection wound dressing.

## AUTHOR CONTRIBUTIONS

**Yuting Luo:** Data curation (equal); formal analysis (equal); investigation (equal); methodology (equal); software (equal); validation (equal); visualization (equal); writing – original draft (equal). **Sen Zheng:** Data curation (equal); formal analysis (equal); investigation (equal); methodology (equal); validation (equal); visualization (equal). **Kun Wang:** Data curation (equal); formal analysis (equal); investigation (equal); methodology (equal); software (equal). **Hangqi Luo:** Data curation (equal); formal analysis (equal). **Huiling Shi:** Data curation (equal); formal analysis (equal). **Yanna Cui:** Formal analysis (equal). **Bingxin Li:** Formal analysis (equal). **Huacheng He:** Conceptualization (equal); funding acquisition (equal); project administration (equal); resources (equal); supervision (equal); writing – review and editing (equal). **Jiang Wu:** Conceptualization (equal); funding acquisition (equal); project administration (equal); resources (equal); supervision (equal); writing – review and editing (equal).

## CONFLICT OF INTEREST STATEMENT

The authors declare no conflict of interest.

### PEER REVIEW

The peer review history for this article is available at https://www.webofscience.com/api/gateway/wos/peer-review/10.1002/btm2.10540.

## Supporting information


**Data S1:** Supporting Information

## Data Availability

Data available on request from the authors.
