# Correction to “Drug cross‐linking electrospun fiber for effective infected wound healing”

**DOI:** 10.1002/btm2.10758

**Published:** 2025-03-17

Luo Y, Zheng S, Wang K, et al. Drug cross‐linking electrospun fiber for effective infected wound healing. *Bioeng Transl Med*. 2023;8(6):e10540. doi:10.1002/btm2.10540

The corrected images are shown below. These errors will not affect the conclusion.

In Figure 5b, on day 4 (D4), we misused the image of TA Solution group for PVA Fiber group.

**FIGURE 5:**
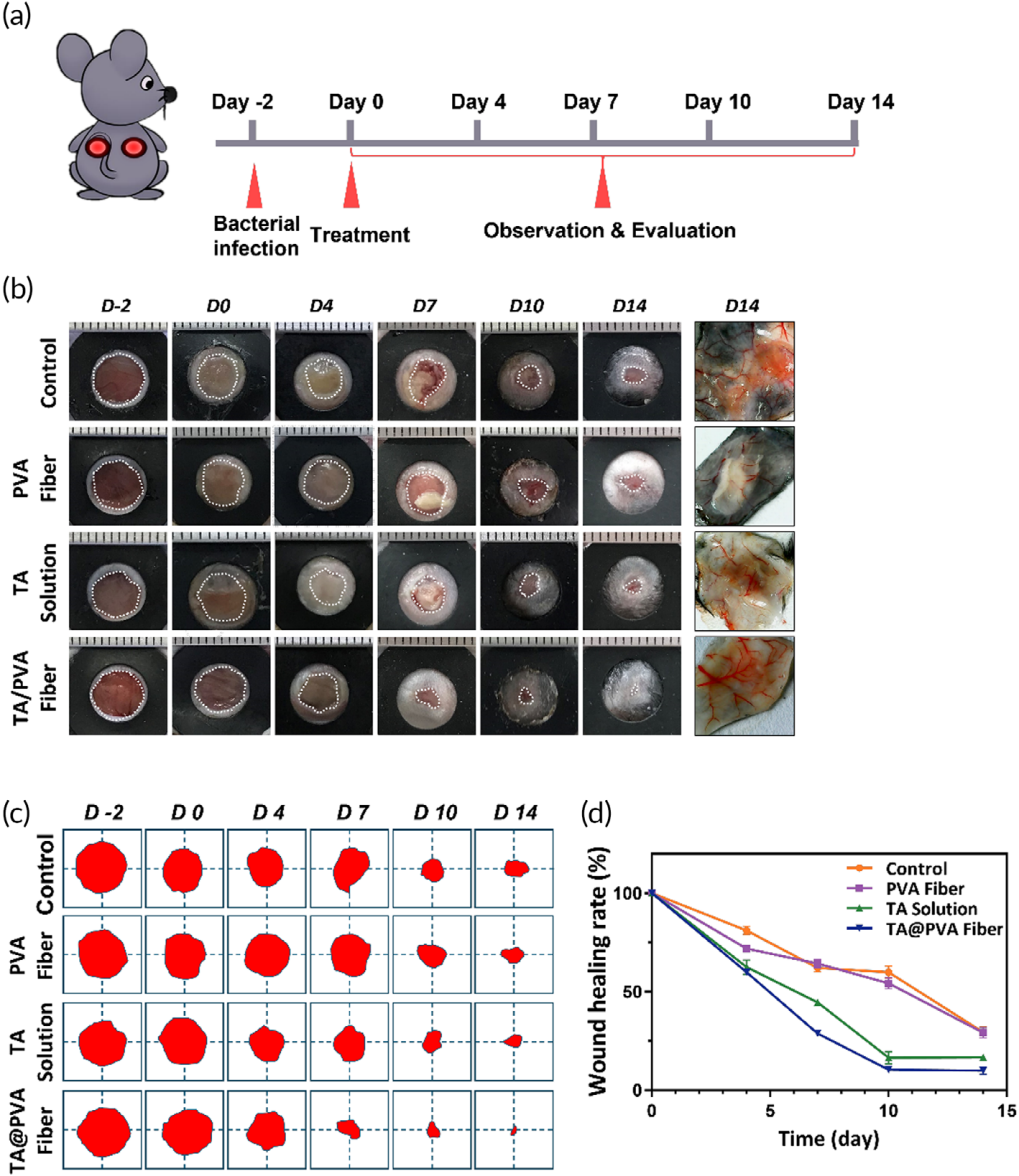
(a) Schematic diagram of the in vivo treatments of phosphate‐buffered saline (PBS (Control)), polyvinyl alcohol (PVA) fiber, tannic acid (TA) solution, and TA/PVA fiber on a mouse model. (b) Representative images of wounds after different treatments. The rightest column: representative dermatoscopic photographs of the skin tissues on Day 14 after different treatments. (c) Wound area traces at different times. The red color area represented the residual wounds. (d) Quantitative analysis of wound areas of different groups.

In Figure 9c, we misused the images of PVA Fiber group for TA/PVA Fiber group.

**FIGURE 9:**
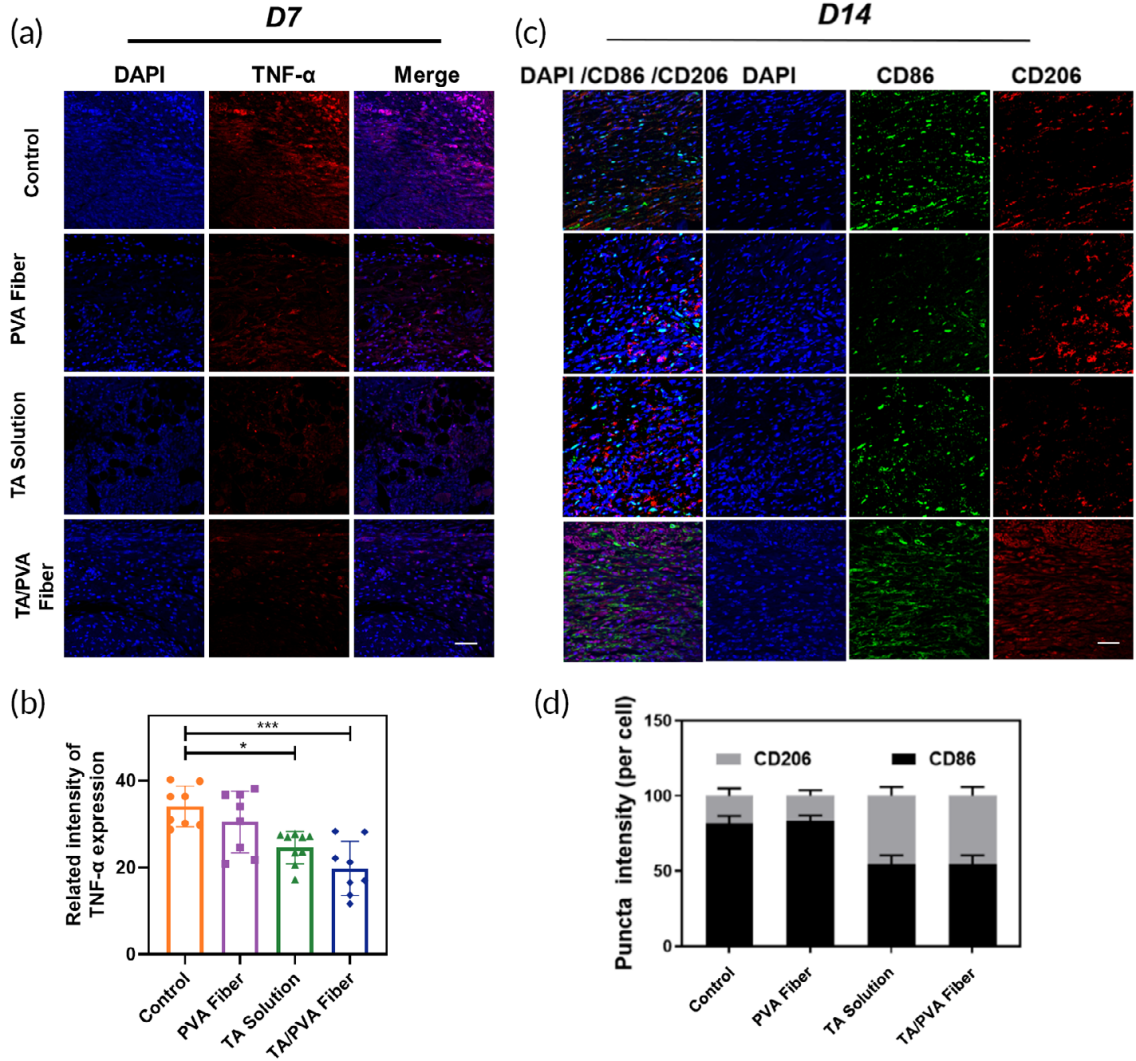
(a) The immunofluorescence staining images of tumor necrosis factor‐α (TNF‐α) (red) of skin wounds on Day 7. The nuclei were stained as blue by 4′,6‐diamidino‐2‐phenylindole (DAPI). Scale bar = 50 μm. (b) Quantitative data of TNF‐α based on the immunofluorescence staining. Data were given as mean ± SD, ***p < 0.001, *n* ≥ 3. (c) Immunofluorescence staining of CD86 (green) and CD206 (red) in wounds with different treatments on Day 14. The nuclei were stained as blue by DAPI. Scale bar = 50 μm. (d) Quantified expressions of CD206 and CD86 in wounds with different treatments on Day 14 (*n* = 3).

In Figure 10, for spleen images, we misused the image of TA Solution group for PVA Fiber group.

**FIGURE 10:**
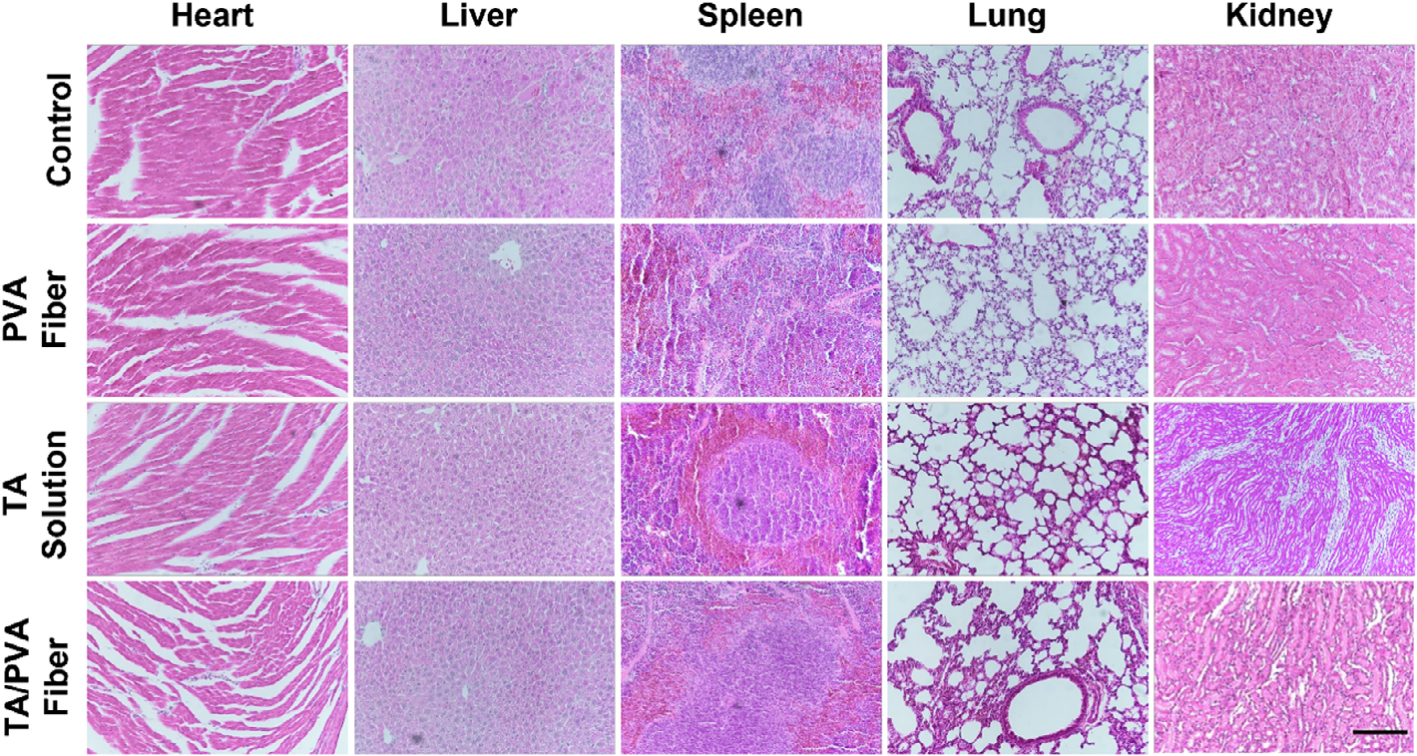
Hematoxylin and eosin (H&E) staining images for major organ tissues on Day 14 after various treatments. Scale bar = 200 μm.

We apologize for these errors.

