# Supplementary material for: Drug cross‐linking electrospun fiber for effective infected wound healing
Source: Bioeng Transl Med. 2023 May 5;8(6):e10540. doi: 10.1002/btm2.10540 (PMC10658581; doi:10.1002/btm2.10540)
Supplement: Supplementary file 1 — Data S1: Supporting Information [file BTM2-8-e10540-s001.docx]

Supporting Information

Drug Crosslinking Electrospun Fiber for Effective Infected Wound Healing

Yuting Luo^1#^, Sen Zheng^1#^, Kun Wang^1#^, Hangqi Luo^1^, Huiling Shi^1^, Yanna Cui^1^, Bingxin Li^2^, Huacheng He^2*^, Jiang Wu^1*^

^1^School of Pharmaceutical Sciences, Key Laboratory of Biotechnology and Pharmaceutical Engineering, Wenzhou Medical University, Wenzhou, Zhejiang 325035, P.R. China

^2^College of Chemistry and Materials Engineering, Wenzhou University, Wenzhou, Zhejiang 325035, P.R. China

*Corresponding authors:

Huacheng He: hehc@wzu.edu.cn

Jiang Wu: woody870402@hotmail.com

# These authors contributed equally to this work.

**Experimental section**

**S1. *In Vitro* swelling studies**

To determine the swelling ability of the obtained fibers, the weight of dried and wet nanoﬁber was compared at speciﬁc time points. Dried nanoﬁbers (1ⅹ1 cm^2^) were weighed and soaked in PBS (pH 7.4) at 37 °C. Weights of the wet nanoﬁbers were recorded at speciﬁc time points until saturation was achieved. Swelling ratio (S) was measured using the following relationship:

$$S(\%)=(W_{s}-W_{d})/W_{d}\times100$$

where W_d_ and W_s_ are the dry and wet weight of the electrospun nanoﬁber, respectively.

**S2. Cytotoxicity of obtained nanofibers**

**MTT assay**: HaCaT cells and NIH 3T3 cells were seeded at a density of 5,000 cells / well in 96-well plates and cultured for 24 h in an incubator (37 ℃, 5%CO_2_). Cells were treated with control, TA solution, extracts of PVA Fiber and TA@PVA Fiber (equivalent to 5 μg/mL TA concentration) for 24 h or 48 h, respectively. And then discard the drug-containing culture media, 100 μL of fresh DMEM and 20 μL MTT were added to each well and incubatd for 4 h in the incubator. Carefully discard the supernatant with a needle. Then 120 μL DMSO solution was added to dissolve formazan at the bottom of the pore and fully shaken, absorbance of the solution was measured at λ = 490 nm.

**Live/Dead staining**: HaCaT cells and NIH 3T3 cells were seeded at a density of 75,000 cells / well in 12-well plates and cultured for 24 h in an incubator (37 ℃, 5%CO_2_). Cells were treated with control, TA solution (5 μg/mL) and extracts of PVA Fiber and TA@PVA Fiber (equivalent to 5 μg/mL TA concentration) for 24 h or 48 h, respectively. Then 500 μL of live/dead dye (containing 500 μL PBS and 0.5 μL Calcein-AM and 0.5 μL PI dye) was added to each well, and incubated for 30 min in a 37 ℃ incubator. Then the fluorescent dye was subsequently discarded and washed several times with PBS in order to reduce background noise. Photographs were recorded under an inverted fluorescence microscope (ECLIPSE, Ts2, Nikon, Japan).

**S3. Antibacterial activity**

The antibacterial activity of the electrospun TA/PVA nanofibers against *S. aureus* was further studied by the agar diffusion method. The inhibition zones around PBS (negative control), TA solution (low and high), gentamicin (positive control) and TA@PVA fiber (low and high) were detected. TA@PVA fibers were cut into round pellicles with a diameter of 10 mm and sterilized by ultraviolet light for 1 h. 100 μL bacteria suspension (about 1 × 10^6^ CFU/mL) was uniformly spread on LB agar plates, and the sterilized samples were carefully placed in the middle of the plates. Zones of inhibition were measured after incubation for 24 h in a 37 °C incubator.

**S4. Immunofluorescence staining**

The wound tissues were further assessed by immunofluorescent staining. All the procedures followed the manufacturer’s instructions. The primary antibodies included anti-CD31 (ab28364, 1:50, Abcam), anti-𝛼-SMA (ab7817, 1:300, Abcam), anti-CD206 (DF4149, 1:200, Affinity), anti-CD86 (501199, 1:200, Zenbio), anti-Ly6G (551459, 1:500, BD Pharmingen) and anti-TNF-α (11948, 1:250, Cell Signal Technology). Tissue sections were incubated with fluorescence secondary antibodies IgG Alexa Fluor® 488, goat anti-mouse IgG Alexa Fluor® 647, Alexa Fluor FITC goat anti-rat secondary antibody and Alexa Fluor FITC goat anti-rabbit secondary antibody, respectively, at 37 °C for 60 min. Finally, the tissues were stained with 4′, 6-diamidino-2-phenylindole (DAPI) (Beyotime, China) for 5 min. The fluorescence images were photographed by a Nikon confocal laser microscope (A1 PLUS, Nikon, Japan). Fluorescent intensities were quantified using Image-Pro Plus software (n > 5).

**
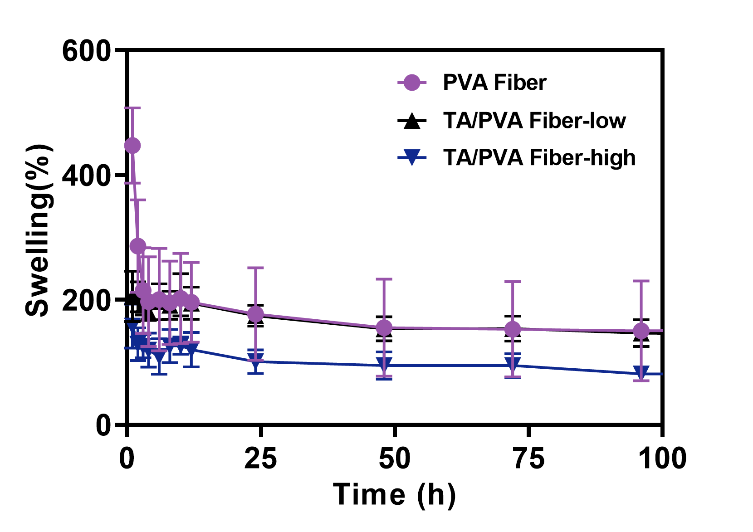
**

**Figure S1.** Swelling behavior of the electrospun fibers


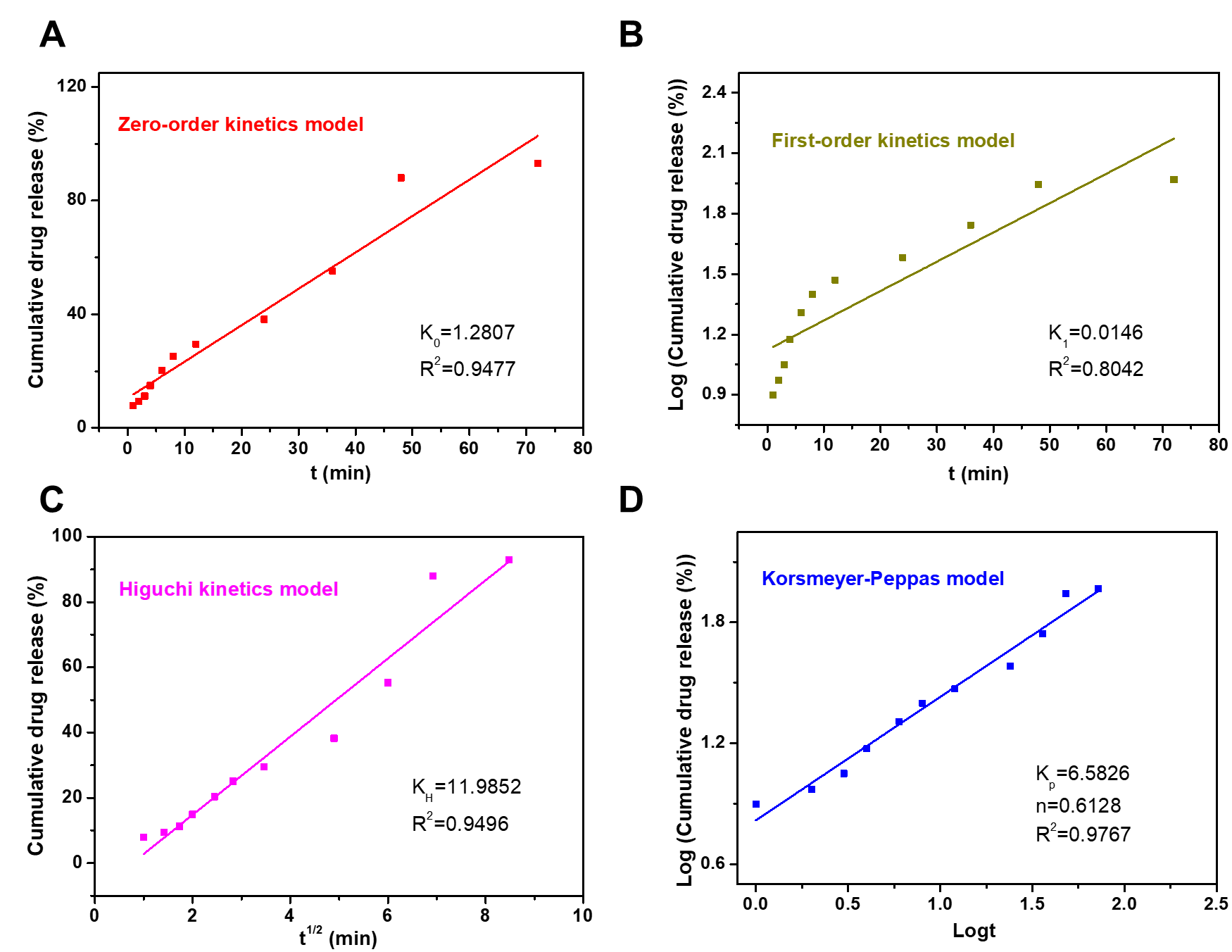


**Figure S2**. The kinetic plots of zero order (A), ﬁrst order (B), Higuchi (C) and Korsmeyer-Peppas (D) models.

**Figure S3**. (A) Schematic illustration of the in vitro study of the degradability of the TA/PVA fiber. PBS (pH7.4) was used as the degradable buffer. (B) Photographs of the TA/PVA fiber in the transwell up to 5 days. Top panel: top view of the fiber. Bottom panel: side view of the fiber. (C) Photographs of the TA/PVA fiber in the wound bed of the mouse model for up to 5 days.


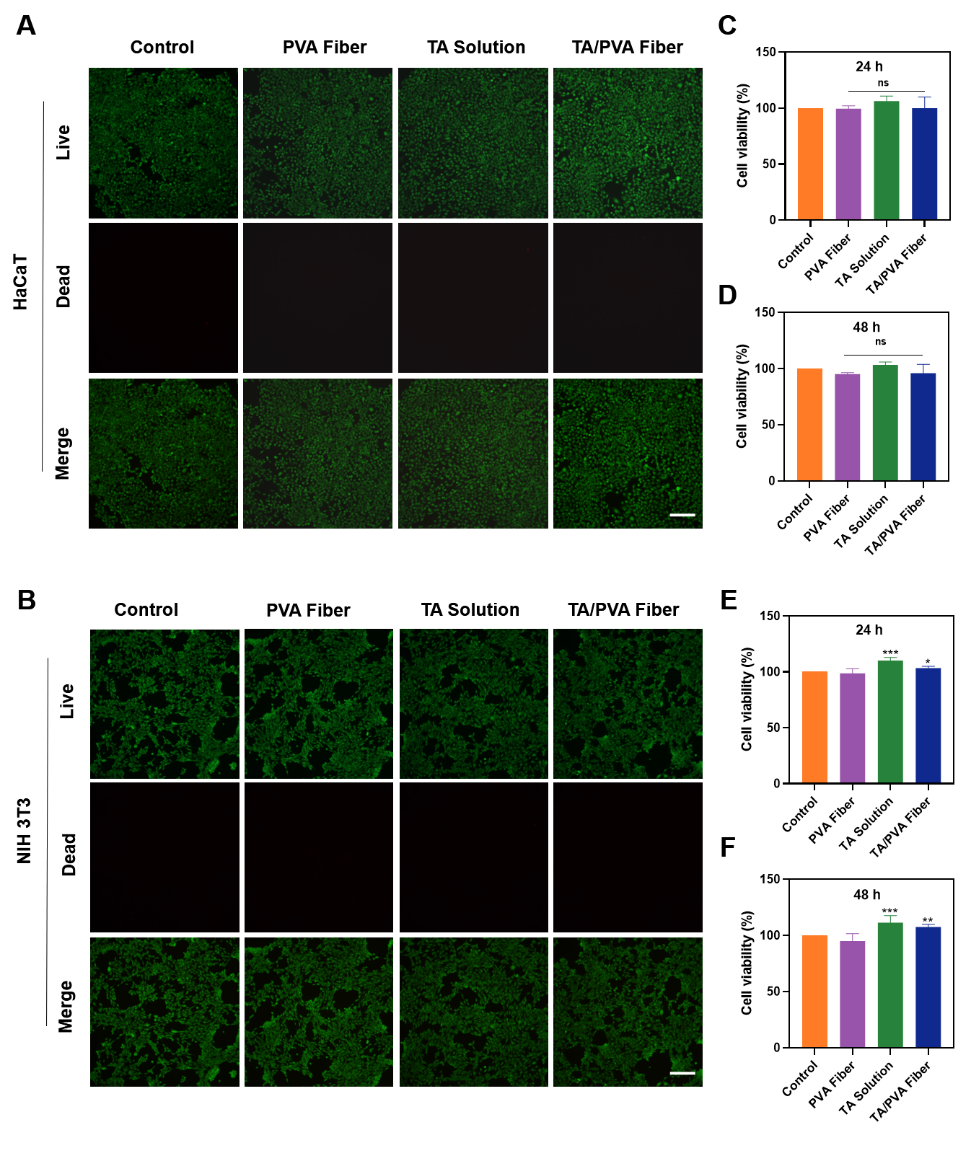


**Figure S4.** Live/dead assay of different treatments in HaCaT(A) and NIH 3T3 (B) cell lines. Cells were treated by PBS, PVA fiber, TA solution, TA/PVA fiber for 24 h. and then the live (green) and dead (red) cells were imaged under a fluorescence microscope. Scale bar = 200 μm. MTT assay of different treatments in HaCaT for 24 h (C) and 48 h (D). MTT assay of different treatments in NIH 3T3 for 24 h (E) and 48 h (F). Data are presented as mean ± standard deviation, * *P* < 0.05, ** *P* < 0.01, *** *P* < 0.001 indicate statistical significance.

**Figure S5**. (A) Gross images of PVA nanofibers implants after 7 days. Cross-sectional images stained by (B) H&E and (C) Masson’s trichrome.


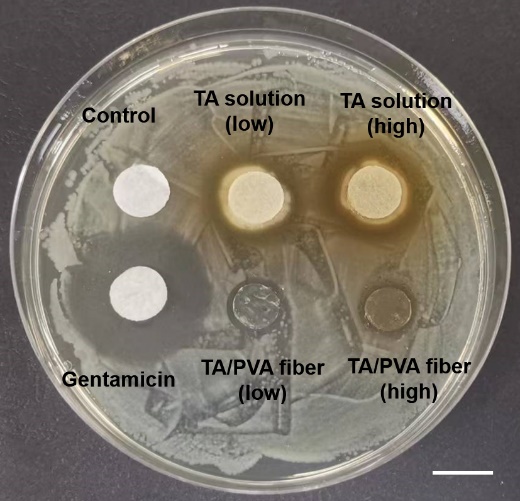


**Figure S6.** Antibacterial activities of the samples against *S. aureus.* Scale bar = 1 cm.


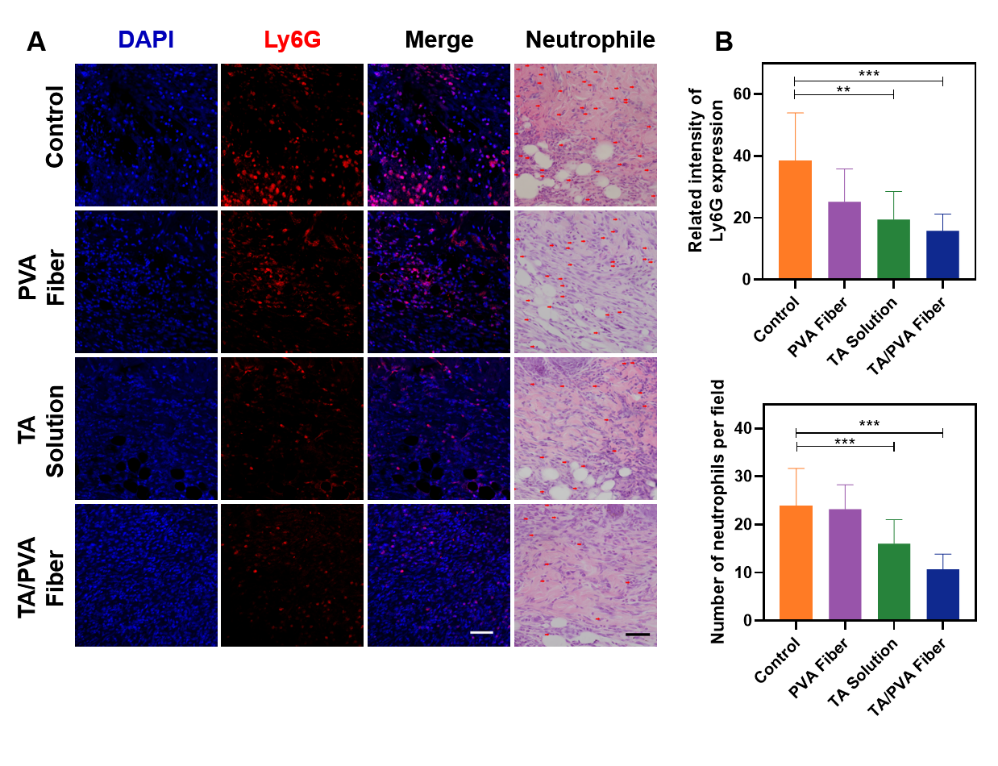


**Figure S7.** (A) Histological analysis of inflammation from H&E staining and immunofluorescence images of Ly6G at the wound site with different treatments (Control, PVA Fiber, TA solution, TA/PVA Fiber) at day 7. Red arrows indicate the location of neutrophils. Scale bar, 200 μm for H&E images and 50 μm for immunofluorescence images. (B) Statistical analysis of Ly6G and neutrophils number. Data are presented as mean ± standard deviation, ** *P* < 0.01, *** *P* < 0.001 indicate statistical significance.
